# Emerging Therapeutic Approaches to Target the Dark Side of Senescent Cells: New Hopes to Treat Aging as a Disease and to Delay Age-Related Pathologies

**DOI:** 10.3390/cells12060915

**Published:** 2023-03-16

**Authors:** Roula Khalil, Mona Diab-Assaf, Jean-Marc Lemaitre

**Affiliations:** 1 IRMB, University Montpellier, INSERM, 34090 Montpellier, France; roula.khalil@inserm.fr; 2 Fanar Faculty of Sciences II, Lebanese University, Beirut P.O. Box 90656, Lebanon; mdiabassaf@ul.edu.lb

**Keywords:** cellular senescence, age-related disorders, SASP, senolytic drugs, immune surveillance

## Abstract

Life expectancy has drastically increased over the last few decades worldwide, with important social and medical burdens and costs. To stay healthy longer and to avoid chronic disease have become essential issues. Organismal aging is a complex process that involves progressive destruction of tissue functionality and loss of regenerative capacity. One of the most important aging hallmarks is cellular senescence, which is a stable state of cell cycle arrest that occurs in response to cumulated cell stresses and damages. Cellular senescence is a physiological mechanism that has both beneficial and detrimental consequences. Senescence limits tumorigenesis, lifelong tissue damage, and is involved in different biological processes, such as morphogenesis, regeneration, and wound healing. However, in the elderly, senescent cells increasingly accumulate in several organs and secrete a combination of senescence associated factors, contributing to the development of various age-related diseases, including cancer. Several studies have revealed major molecular pathways controlling the senescent phenotype, as well as the ones regulating its interactions with the immune system. Attenuating the senescence-associated secretory phenotype (SASP) or eliminating senescent cells have emerged as attractive strategies aiming to reverse or delay the onset of aging diseases. Here, we review current senotherapies designed to suppress the deleterious effect of SASP by senomorphics or to selectively kill senescent cells by “senolytics” or by immune system-based approaches. These recent investigations are promising as radical new controls of aging pathologies and associated multimorbidities.

## 1. Introduction

It is definitely clear that living longer is a leading risk factor for increasing vulnerability to the development of the most prevalent pathologies, including cardiovascular diseases, neurodegenerative diseases, and cancer [1]. Increasing longevity presents a huge challenge for medical care, as aging progression is diverse and is not absolutely correlated to chronological age. Although a preventive medicine would have been the most suitable to prevent age related disease, pertinent biomarkers were not available to precisely measure precisely physiological age. However, new insights into the cellular and molecular events playing a role in biological aging led to the definition of a restricted set of biomarkers as potential targets to fight tissue aging deteriorations [2,3]. One emerging factor is the accumulation of senescent cells in tissues and organs, now considered as a common denominator in a vast majority of age-related pathologies [4,5]. Senescent cells are especially abundant at sites of age-related pathologies, and a great deal of evidence from mouse models demonstrated a causal role of senescent cells in several age-related diseases, such as sarcopenia, lordokyphosis, cataracts, osteoporosis, aged hematopoietic system, vasomotor dysfunction, atherosclerosis, neurodegeneration, idiopathic lung fibrosis, osteoarthritis, hepatic steatosis, and hair loss [6,7,8,9,10,11,12,13,14], demonstrated by genetic approaches the clearance of p16-expressing senescent cells in mice models [15,16]. As this approach gave rise to a global delay of the onset of deterioration of several organs and the appearance of the corresponding age-related diseases, it led to a new paradigm considering aging as a disease and not only as a biological process. Targeting senescence seems to be a promising approach for treatment and prevention of aging to further promote an increased health longevity [17,18].

The fundamental characteristic of senescent cells is an irreversible cell cycle arrest that occurs in normal proliferating cells in response to various forms of cellular stress. Telomere shortening, UV radiation, increased levels of ROS species, oncogene activation, direct DNA damage, and other forms of stress that elicit activation of the DNA damage response pathway can lead to senescence [19,20,21,22]. Senescence was originally understood as a physiological cellular response aiming to prevent propagation of damaged cells in the organism [4,23,24,25,26,27,28,29]. Later, a number of additional beneficial functions, such as tissue repair, wound healing, and embryonic development, were discovered [5].

In addition to the protective role of cellular senescence, the long-term presence of senescent cells was revealed to be detrimental to the organism [5,30]. Indeed, the proliferative incompetency of senescent cells is accompanied by a resistance to apoptosis, as well as the secretion of a complex plethora of factors, referred to as the senescence-associated secretory phenotype (SASP). It contains, chemokines, cytokines, tissue damaging proteases and growth factors, and proinflammatory factors that normally assist in their removal by the immune system [31,32]. Studies on diverse animal models indicate that multiple components of the immune system, including NK cells, T cells, and macrophages, are involved in controlling the presence of senescent cells in tissues [26,33,34,35,36,37]. The efficacy of this removal is variable among tissues and pathological conditions, and the mechanisms and rules regulating the homeostasis of senescent cells are yet to be fully understood. In the elderly, senescent cells and their SASP increasingly accumulate in tissues and contribute to the establishment of a chronic inflammation, so called “inflammaging”, due to continuous secretion of proinflammatory cytokines [24,38,39] considered as a feature of the majority of age-related diseases [40].

In the last decade, ground-breaking studies have provided proof of concept that either relieving the burden of senescent cells or suppressing SASP can have a beneficial impact to age-related tissues alterations, thus ameliorating the health state of an organism and subsequently increasing life span [16,41]. These studies provide insights for the development of novel therapeutic strategies to target senescent cells, called senotherapies. Senotherapies include the discovery of small molecules acting as selective eliminators of senescent cells (senolytics) or as inhibitors of the SASP and of the signaling pathways that control it (senomorphics). This opened the way to an unexpected new field of research aiming at the development of senotherapeutic strategies that can interfere with and delay the aging process. The underlying theme of this review is the recent and rapid development of the various and complementary senotherapeutic approaches to alleviate the impact of senescent cell in aging. We reflect on the medical development of senescence-focused procedures, and the implications for future opportunities and challenges to fight aging diseases, including their limitations, efficacy, and safety.

## 2. Senescence in Physiological Processes

**Figure 1:**
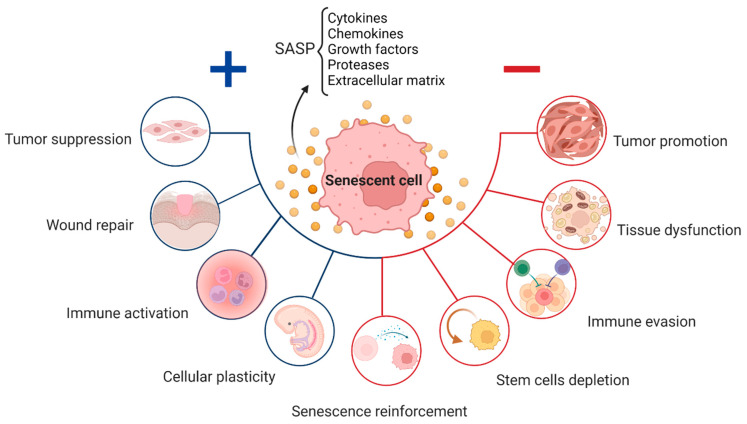
Role of senescent cells throughout life: positive (blue) and negative (red) effect depending on the level of senescence and SASP components released, including cytokines, chemokines, and other molecules, which affects the neighboring cells.

Despite the detrimental effects of persistent cellular senescence and SASP being widely documented, these systems play crucial and appropriate roles in normal physiological processes, such as embryogenesis, wound healing, and fibrosis (Figure 1) [4,25,42,43]. Senescence is essential for the healthy functioning of several physiological processes, including embryonic development, wound healing, and fibrosis. This kind of senescence, also known as “acute senescence” [5], appears to be strictly controlled; it only affects a certain subset of tissue cells, which are then quickly eliminated by the immune system.

Cellular senescence is carefully regulated during embryogenesis, starts at certain periods and sites, and is involved in morphogenesis, tissue remodeling, and cell population balance [42,43]. Embryos at mid to late stages of development display senescent cells in very specific anatomical structures and locations throughout the embryo. Positive areas include the developing limbs, the tip of the tail, the otic and brain vesicles, the fusing sternum midline, the gut endoderm, and the closing neural tube. In each of these cases, senescent cells must be efficiently removed by macrophages to allow proper tissue formation and maturation. This is important, as suppression of cellular senescence in mice results in developmental abnormalities, highlighting the crucial function of cellular senescence in proper embryogenesis [4].

The expression of p21 and other SASP components are two characteristics that embryonic senescent cells have in common with oncogene-induced senescent cells. The absence of p16 expression, DNA damage, and the release of IL-6 and IL-8 are some other variations between adult and embryonic senescence [43]. Embryonic senescence appears to be solely dependent on p21, indicating that it is not affected by p53, DNA damage, or other cell cycle inhibitors. The senescent cells in the embryo occur for a short period of time, undergo cell death, and are then eliminated by the immune system before birth [42,43]. As a result, throughout embryo development, TGF-β activates SMAD, which works in conjunction with FOXO proteins to regulate p21 expression and p21-mediated cell cycle arrest.

Later in life, cellular senescence is initiated during the first phases of tissue repair, suggesting that wound healing and tissue regeneration depend on a similar process of cellular senescence as seen during embryonic development [25,42].

Senescent fibroblasts and endothelial cells produce many proteins, including PDGF-AA, upon injury [25]. To begin wound healing, PDGF-AA stimulates myofibroblast differentiation and granulation tissue formation. Additionally, SASP-associated proteases aid in regulating excessive fibrosis [25]. Besides this, pharmacological cellular senescence suppression in vivo decreases wound healing [16,25]. Thus, the importance of cellular senescence in development and repair is highlighted by the necessity of senescent cells and SASP at the early stages of tissue remodeling [4].

It is important to point out that the development of tissue fibrosis has been shown to benefit from cellular senescence. Senescence has also been proposed to reduce fibrosis through the production of proteases during wound healing [25]. However, in the lung, it is considered to induce pulmonary fibrosis [25,44].

Within several days of a skin injury, senescent fibroblasts and endothelial cells emerge at the wound site; lack of senescent cells results in delayed wound healing. Treatment with recombinant PGDF-AA can prevent this delay, but severe fibrosis can still occur [25]. This, in turn, is limited by senescence of myofibroblasts, which, when not senescent, promote fibrosis by producing ECM. Aside from this, senescent cells release ECM-degrading proteases into the SASP [45].

Similarly, in the presence of a chronically damaged liver with severe fibrosis, aging of hepatic ECM-producing stellate cells can inhibit fibrosis, just as it does in wound healing (HSC). Active HSCs, as they become senescent, decrease ECM release and improve immune surveillance. HSC senescence can be triggered by insulin-like growth factor-1 (IGF-1) or CCN1, as well as by the anti-inflammatory factors IL-10 and IL-22, through the signal transducer and activator of transcription 3 (STAT3) and p53-dependent pathway.

During regeneration in salamanders, a large number of senescent cells were found in the limb. However, when the limb had fully recovered, these senescent cells disappeared [46]. Other experiments revealed that, when both normal and senescent cells were inserted into a salamander, the senescent cells also disappeared rapidly. This suggests that senescent cells could potentially be involved in the regeneration process and that salamanders possess a functional system capable of effectively clearing these cells. Macrophages, among other immune system components, may help in the removal of senescent cells from some tissues, as shown by previous studies. Demonstrating how senescent cells were surrounded by macrophages in living salamanders regenerated limbs supported this theory. Similarly, senescent cells were not eliminated as efficiently in some salamanders whose macrophages were destroyed by the toxic clodronate-loaded liposomes, as in salamanders with active macrophages [47]. Therefore, macrophages are a vital factor in the process of removing senescent cells from salamander tissues, and this mechanism of eliminating senescent cells may unlock the secret of the salamander’s ability to maintain regeneration during aging.

Because senescence play an important role in physiological processes, such as wound healing [25], tissue remodeling, and embryonic development [42,43], the emerging approaches of clearing senescent cells to ameliorate age-related tissue deteriorations have been questioned.

## 3. Senescence in Age-Related Diseases

Aging is the largest risk factor for developing many disorders, ranging from tissue degeneration to cancer. One pivotal driver of most of the major age-related pathologies is cellular senescence [2,5]. There are different types and mechanisms of senescent cells formation. Cellular senescence, as first described by Hayflick and Moorhead, referred to the finite lifespan of primary human fibroblasts in culture [48,49]. This observation describes only one particular type of cellular senescence that is caused by the attrition of telomeres, called replicative senescence (RS), a naturally occurring process that takes place every cell division [50]. Cellular senescence is induced by a variety of stress-causing, irreversible damages. These can include oncogene signaling, DNA damage, irradiation, or chemotherapy, causing the activation of tumor suppressor networks, including p53, p16, and p21, as well as cell cycle arrest [22,24,30,31,51]. In this case, it is categorized as a premature senescence. Thereby, senescence acts as intrinsic tumor suppressive mechanism that might prevent the proliferation of damaged cells. Irrespective of the stimulus, the senescence program is executed upon persistent activation of p53/p21 and/or RB/p16 tumor suppressor pathways, orchestrating the transition to and the maintenance of the senescent phenotype [24].

## 4. The Various Types of Senescence

### 4.1. Replicative Senescence

In human cells, the cell cycle arrest elicited by continuous culture is called replicative senescence in response to telomere attrition around an average telomere length of 6–8 kbp. A senescent cell likely contains one (or a few) much shorter telomeres [52], and these cells are characterized by absence of proliferation, but they possess long-term viability.

**Figure 2:**
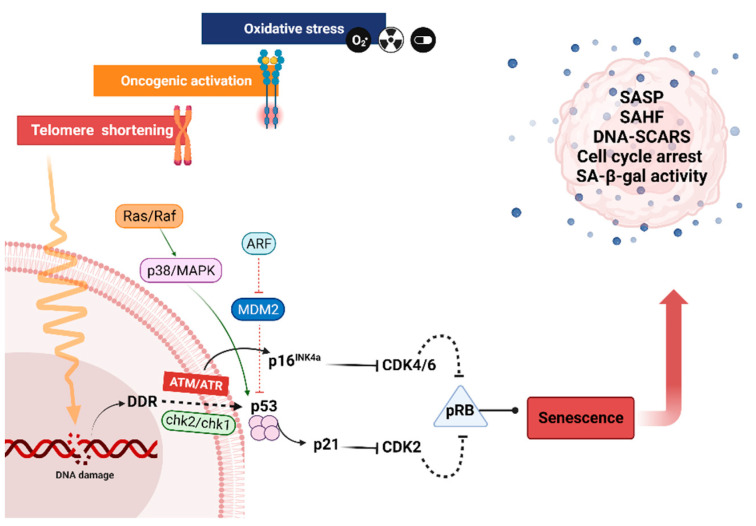
General overview of the different cellular senescence induction pathways and related characteristics. Three different types of senescence: replicative senescence, oncogene-induced senescence, and stress-induced senescence involving persistent DNA damage response (DDR).

Telomeres are composed of TTAGGG sequence repeats at the ends of chromosomes, which preserve the genetic material. The length of the non-replicated DNA molecules decreases with each cell division. Thus, in highly proliferating tissues or in telomere syndromes, progerias, and premature aging, which include hereditary diseases linked to early senescence, the telomeres are shortening much more quickly [53]. Telomere attrition is often considered to be a DNA damage and a DNA damage response (or DDR) [54] occurring after approximately 60–70 cell divisions for embryonic fibroblasts [48] (Figure 2). Ataxia telangiectasia mutated (ATM), ataxia telangiectasia, and Rad3-related (ATR) are the first checkpoint kinases that become active in response to DNA damage [55]. Subsequently, these kinases cause the activation of various proteins, including checkpoint kinase 2 (Chk2). ATM phosphorylation fully activates Chk2 after triggering oligomerization and autophosphorylation of Chk2, which in turn transmits DNA damage signaling to several components involved in cell cycle arrest, apoptosis, and DNA repair, including phosphorylation and activation of the tumor suppressor protein p53, which initiates p21 expression [5,56,57]. P21 inhibits cyclin-dependent kinase 2 (CDK2), which prevents the phosphorylation of the retinoblastoma protein (Rb). As a result, transcription of the proteins needed for the subsequent cell cycle stage (S Phase) is prevented, resulting in cell cycle arrest at the G1 phase [5,57]. Along with the Chk2-p53-dependent regulation of p21, cancer cells implement a p53-independent mechanism involving retrovirus-mediated expression of Chk2 kinase to transcribe p21 without requiring p53 protein [56]. Moreover, consistent steps occur for human diploid cells that have gone through RS, where in the later passages, when cells lose their ability to proliferate and become senescent, p21 protein expression first rises. Later, the levels of p21 drop as those of p16 mRNA and ultimately its p16 protein steadily rise. The protein p16, also known as cyclin-dependent kinase inhibitor 2A, or CDKN2A, slows down cell division by delaying the transition from the G1 phase to the S phase of the cell cycle. This protein, with CDK4 and CDK6 kinases, form a complex that blocks the phosphorylation of Rb, making p16 a final key factor in stabilizing senescence, which is the main reason why cancer cells frequently have mutations in p16 rather than in p21 [58]. Because senescence is a permanent cell cycle arrest in G1 phase, it must be permanently maintained either by the previously described DDR, which can be prolonged or permanent [59], or by the development of senescence-associated heterochromatin foci (SAHFs), which prevent transcription of E2F-regulated target genes required for cell proliferation. However, senescence can also be observed in the absence of SAHFs, for example in cells that have become senescent following loss of PTEN or activation of protein kinase B (AKT), or in murine cells that do not develop robust SAHFs [60,61].

### 4.2. Premature Senescence

Premature senescence is the second category of senescence initiated by various stress, independently from telomere shortening [62,63], either through a DNA damage response-dependent or independent pathway (Figure 2). Different stress factors and cell types can vary the mechanisms of premature senescence. This senescence category includes the oncogene-induced senescence (OIS) and stress induced senescence (SIS), oxidative stress or PTEN-induced senescence (PICS), or therapy-induced senescence (TIS) as examples further detailed hereafter.

OIS, a different type of senescence, is caused by the downregulation and upregulation of certain oncogenes, often in combination with other changes [63]. Primary fibroblasts, in association with the accumulation of p53, p21, and p16 proteins, become senescent due to Ras oncogene (Figure 2) [22]. In addition, Ras activation leads to senescence of human primary thyrocytes, which arise from the p16 pathway activation via an interaction between IL-8 and the C-X-C motif chemokine receptor 2 (CXCR2), also known as interleukin-8 receptor beta (IL-8RB) [64]. Taking the example of the non-cancerous thyroid cell line, H-RasV12 initiates senescence with higher expression of p21 and absence of p16 induction [65]. This led to the hypothesis that, while p16 is necessary for the maintenance of senescence and is increased subsequently, p21 plays a crucial initial function in the induction of senescence and is first increased before declining [58,66]. H-RasV12, on the other hand, was discovered to control the H_2_O_2_-producing NADPH oxidase system NOX4-p22^phox^ in the non-cancerous thyroid cell line. The fact that this complex was found in the nuclear membrane and endoplasmic reticulum suggests that it functions as a DNA-damaging agent. Therefore, replicative stress brought on by a persistent oncogenic signal that upregulates the DNA replication protein CDC6 or ROS buildup might result in DNA damage [65]. Ras-induced abnormal proliferation can also cause the dysregulation of E2F1, activation of ARF expression, restoration of p53, and apoptosis [67]. Senescence, particularly OIS, is a cancer-fighting mechanism, as demonstrated by melanocytes, where natural senescence in cell culture is p16-dependent. In vivo, p16-dependent cell senescence occurs to some extent in the common benign melanocytic naevi. This proposes a plausible method by which melanoma following a mitogenic mutation, such as the NRAS or BRAF mutations, are repressed by the p16/Rb pathway, validating the hypothesis that senescence is a very effective defense against cancer [68].

Premature senescence is triggered by overexpression of the MAPK/ERK kinase 1 (MEK1), which stimulates ERK1/2 in human non-immortalized intestinal epithelial stem cells, but the same intervention produces mitogenesis in immortalized mouse intestinal stem cells. MEK1 constitutive activation promotes senescence in primary murine fibroblasts, but it induces mitogenesis in cells lacking p53 or p16. These two divergent outcomes depend on the integrity of a senescence pathway mediated by p53, p21, and p16. Hence, p53 or INK4A/ARF mutated cells can bypass senescence. P16 silencing is the most relevant molecular alteration in cancer and cell immortalization [67]. Similarly, it has been found in primary murine fibroblasts deficient in p53 or p16, where senescence was prevented by activation of the Ras oncogene protein [22]. Senescence can be produced not only by the MEK1 pathway, but also by ectopic expression of mitogen-activated protein kinase phosphatase 2 (MKP2), which is involved in ERK nuclear signaling. Inactivation of ERK2 by MKP2 is also present during RS, and elimination of MKP2 expression or expression of phosphatase-resistant ERK2 can delay it [69].

PTEN loss-induced senescence (PICS) is a form of senescence in which PTEN negatively regulates the PI3K/AKT pathway. PTEN deficiency causes PICS without initiating DDR via the PI3K/AKt/mTOR-p53-p21 signaling pathway. Mammalian rapamycin target complexes 1 and 2 (mTORC1 and mTORC2) interact with the mouse double minute 2 (MDM2) homolog to control p53 by direct phosphorylation and, subsequently, accumulation of p21 induces senescence [70]. PICS is convenient for pro-senescent cancer therapy, as it is not followed by typical hyperproliferation or DDR. In a human prostate cancer xenograft model, pharmacologically activated PICS through PTEN inhibition induced senescence and prevented carcinogenesis [71].

Both ROS and oxidative stress are very important for senescence and are frequently found in senescent cells. Senescence requires the use of two signaling pathways, p21 controlling cell cycle arrest and AKT increasing ROS in p53-dependent premature senescence. p53 activates mTORC2 and triggers the activation of AKT, which in turn controls intracellular ROS levels by directly binding and activating the nuclear factor kB (3) promoter to the NADPH oxidase 4 (NOX4) promoter. Nevertheless, p21 can also increase ROS levels, depending on the cellular environment. In the absence of p53, it is plausible that p21 promotes both cell cycle arrest and increased ROS generation, but in the case of p53, it only causes cell cycle arrest [72].

In a high glucose environment, senescence can also be triggered. As mouse microvascular endothelial cells are cultured in a high-glucose environment, there is an increase in ROS generation and a decrease in sirtuin 1 (SIRT1) expression, leading to higher acetylation of FOXO1 and p53 and, thereby, to overexpression of p21, resulting in senescence [73].

Additionally, senescence can be promoted by ATM via the lysosomal/mitochondrial axis by preventing the formation of two vacuolar ATPase proton pump domains (V1 and V0), thereby producing insufficient lysosomal acidity and dysfunction of the lysosome/autophagy system. This is exacerbated by ROS accumulation, which leads to mitochondrial dysfunction and ROS-mediated damage, both of which can lead to senescence [74]. The SASP component interferon γ (IFN-γ) is thought to cause senescence in human umbilical vascular endothelial cells (HUVECs) as a result of DNA damage, along with increased ROS production, which may be generated by mitochondria or NADPH oxidase, according to the cell type. Other IFNs have been reported to induce senescence in various cell types [75].

It is worth mentioning that the mechanisms generating premature senescence are not numerous. Some pathways have been identified as being represented in different populations of senescent cells, such as inhibition of IMMP2L in RS or OIS [76], and ROS production appears to be not only a feature of senescent cells [60,77], but can also initiate a senescent phase on its own [74,75].

Another type of premature senescence is TIS, a condition that can be caused by a variety of clinically used drugs, most commonly chemotherapy. Docetaxel, paclitaxel, cyclophosphamide, bleomycin, vincristine, doxorubicin, cyclophosphamide, and cisplatin are a few examples, in addition to radiotherapy, which can induce senescence [63,78,79]. The processes that cause senescence differ and are generally not fully explained. Busulfan, for example, is an alkylating drug used in chemotherapy that, although it can induce DNA strand damage by DNA cross-linking, causes senescence by a p53-independent mechanism triggered by the Erk-p38 MAPK pathway (Figure 2). This mechanism is most likely triggered as a result of increased oxidative stress. The major mechanism of busulfan metabolism is thought to be GSH-S-transferase-catalyzed conjugation of glutathione (GSH), resulting in GSH deficiency and early oxidative stress. This is further supported by increased NADPH oxidase and ROS generation [66].

## 5. Features of Senescent Cells

One of the major challenges in studying cellular senescence is the lack of a clear, sensitive, and accurate biomarker to detect it. The identified hallmarks of senescent cells are not equally prevalent in all senescent cell types, and some of them are not specific to senescent cells. Identification of senescence is difficult due to the underlying biological complexity, and many biomarkers have been used for this purpose. The most robust include higher expression levels of the endogenous CDK inhibitors p16 and p21, greater activity of the lysosomal enzyme senescence-associated-galactosidase (SA-Gal), and buildup of lipofuscin, a un-degradable aging byproduct [80]. Hence, senescent cells are defined by a mixture of these traits [62].

### Morphology and Cell Cycle Arrest

In addition to the irreversible cell cycle arrest, senescent cells undergo morphological and functional alterations, resulting in distorted cellular interactions with surrounding environment [77]. A fluorescent dye, such as DAPI, is used to label the DNA for morphology inspection, enabling visualization and analysis of the nucleus size, being bigger in senescent cells, as well as the fluorescence intensity associated with the DNA per cell nucleus. Supplementary immunocytochemical detection of p14, p16, p21, and p27 markers, none of which are particularly specific, can be carried out [60] in conjunction with the assessment of cell expansion [81]. An additional BrdU or EdU incorporation assay [13,60] and cell counting kits can be used to examine cell cycle arrest [82].

SASP, senescence associated β-galactosidase activity (SA-β-Gal), resistance to apoptosis through pro-survival pathways, and other characteristics are some of the most essential aspects of senescent cells. Therefore, upon visual and cell growth examination, it is advisable to screen cell cultures for two or more of the following putative senescence biomarkers: SA-β-Gal enzyme activity, up-regulation of p16, SAHFs, and DNA-SCARS (DNA damage markers) [60].

However, it is now well known that cellular senescence is responsible for the development and progression of a wide range of major age-related diseases. Long-term accumulation of senescent cells has been associated with neurological and metabolic diseases, cancer, cardiovascular dysfunction, skin, bone, and liver problems, and infections [83]. Although senescent cells are usually cleared by the immune system, considerable numbers of these cells are consistently found in affected areas. The detrimental effects of senescent cells spread to the surrounding tissue environment through a variety of mechanisms, the majority of which are triggered by SASP secretome-associated signals, including homeostatic abnormalities, chronic inflammatory responses, stem cell niche suppression, fibrosis, angiogenesis, induction of epithelial–mesenchymal transition, and cancer cell survival and proliferation conditions [5].

## 6. Senescence Associated β-Galactosidase Activity

SA-β-Gal activity is a key property of senescent cells, and it is also one of the most widely used biomarkers for detecting senescent cells. Having an acidic pH (4.0–4.5), close to that of the natural lysosomal environment, β-D-galactosidase (β-Gal) is a eukaryotic hydrolase cleaving β-linked terminal galactosyl residues from natural carriers, such as gangliosides, glycoproteins, and glycosaminoglycans.

Dimri et al., in 1995, discovered β-Gal activity in senescent human fibroblasts at pH 6, which is different from the pH level at which β-Gal activity is discovered in normal cells, at pH 4. It has been shown that the enzyme at pH 6 is selective for senescent cells and that the elevated activity of β-Gal is due to the upregulation of lysosomal β-Gal activity [38]. Evidence has been demonstrated that the presence of β-Gal in senescent cells is due to an accumulation of the lysosomal enzyme and its subsequent activity at sub-optimal pH. As a result, nonsenescent cells showing reduced lysosomal expression of β-Gal are not detectable, as β-Gal activity is reduced by 99% at pH 6 [84].

At the gene expression level, the origin of SA-β-Gal, which is expressed from the GLB1 gene encoding lysosomal β-D-Gal, was also verified. It can be detected by chromogenic (X-Gal), fluorescent (C12FDG, MUG), or chemiluminescent (Galacton) substrates. Different applications are required depending on the type of substrate. Adherent cell cultures and tissues are needed for X-Gal. Fluorescent substrates, on the other hand, are made from supernatant or cell culture extracts. In addition, cell culture extracts are used in Galacton technology [60].

For the first time, real-time imaging of senescence in tumors with DNA damage was published in 2019. The researchers created a near-infrared (NIR) activatable molecular probe featuring far-red excitation (680 nm), NIR emission (708 nm), and a high “on” ratio following SA-β-Gal activation. Since the electron donating capacity of its oxygen atom is reduced due to glycosylation, a hemicyanine derivative was chosen as the fluorophore. Accordingly, the NIR probe fluorescence for β-Gal (NIR-BG) is low. Upon cleavage of galactose by SA-β-Gal, hemicyanine regains its fluorescence by recovering its zwitterionic resonance state. Then, flow cytometry or confocal microscopy can detect the fluorescence [85]. NIR-BG was then improved to a self-immobilizing and self-igniting NIR fluorescent probe (NIR-BG2). Due to the production of a quinone-methide electrophilic intermediates (pQMs), which binds to intracellular proteins, NIR-BG2 has superior imaging performance and prolonged retention [86].

## 7. Senescence Associated Secretory Phenotype

SASP is another important biomarker of senescence that can be measured by an ELISA assay for a single component at a time. Proteomics analysis, mRNA profiling, multiplex assays, and antibody arrays, in contrast, allow for the simultaneous detection of multiple variables, but they all have a higher cost per sample and are restricted to fewer samples, making ELISA the first choice. Quantitative proteomics based on stable isotope labeling by/with amino acids in cell culture (SILAC) is another technique that can be applied [60]. Senescent cells produce a distinct secretory phenotype known as the SASP, comprising numerous chemokines, cytokines, growth factors, proteases, lipids, and insoluble intracellular macromolecules. The essential SASP is composed of transforming growth factor β (TGFβ), interleukin-1β (IL-1β), IL-1α, IL-6, IL-8, C-C motif chemokine ligand 20 (CCL20), C-X-C motif chemokine ligand 4 (CXCL 4), monocyte chemoattractant protein-1 (MCP1), plasminogen activator inhibitor-1 (PAI1), matrix metalloproteinase 3 (MMP3), prostaglandin E2 (PGE2), and several more. There are some elements of the SASP that are more prevalent than others, but in general they are highly environment-dependent and differ among senescent cells, according to the senescence inducers and the cell types [87,88,89,90].

Senescent cells may operate in a paracrine or autocrine manner through SASP, causing a pro-inflammatory microenvironment that functions in embryonic development, promotes clearance of senescent cells through stimulation and recruitment of immune cells, and to some extent enhances drug resistance and angiogenesis [89]. It is also possible for SASP to propagate senescence and transmit it to surrounding cells [91]. Despite senescence being a protective mechanism against cancer, senescent cells can also stimulate carcinogenicity via the SASP. Thus, SASP may exhibit both a tumor-promoting effect via vascularization and cellular proliferation, or an anti-tumorigenic effect via senescence and immune clearance [63], where effects may depend on the status of p53. Senescent stellate cells in the liver, for example, typically support tumor suppressor M1 macrophages, but in the absence of p53, they promote pro-carcinogenic M2 macrophages. Likewise, loss of p53 in the colon causes an increase in tumor necrosis factor-alpha (TNFα), which leads to tumor cell expansion and proliferation [89]. At the same time, SASP is closely related to inflammation, and thus senescent cells generating SASP play a key role in chronic age-related inflammation of adipose tissue. Adipose tissue is the largest component in human bodies and contributes to higher circulating cytokines, whereas persistent sterile inflammation is an aging hallmark and is strongly linked to aging-associated disorders [92], supporting the relationship between senescence and aging. The progression of SASP is generally in three stages: a rapid phase associated with DDR, an early self-amplifying phase, and a delayed mature phase. Once cell injury occurs, within 36 hours, a G1 phase cell cycle arrest occurs, followed by rapid secretion of TGF-β. Given that DNA damage is almost always present in senescent cells, this phase of DDR may persist, then a few days after the onset of senescence, an initial SASP occurs with low factors levels such as IL-1α and leads to more SASP synthesis via the first self-amplification autocrine loop, which progressively accumulates with time and reaches SASP saturation after four to ten days from the damage [93]. SASP components, such as IL-1α and IL-1β, which are also among the first expressed components, drive autocrine loops, which constitute a key aspect of SASP production [93]. Additionally, several aspects can control the expression and production of SASP, the most important of which are the NF-κB pathway, the p53 pathway, the CCAAT/enhancer-binding protein (C/EBP) pathway [89,94] and the GATA binding protein 4 (GATA4) pathway [89].

### 7.1. SASP Regulatory Pathways

#### 7.1.1. NF-κB Regulatory Pathway

**Figure 3:**
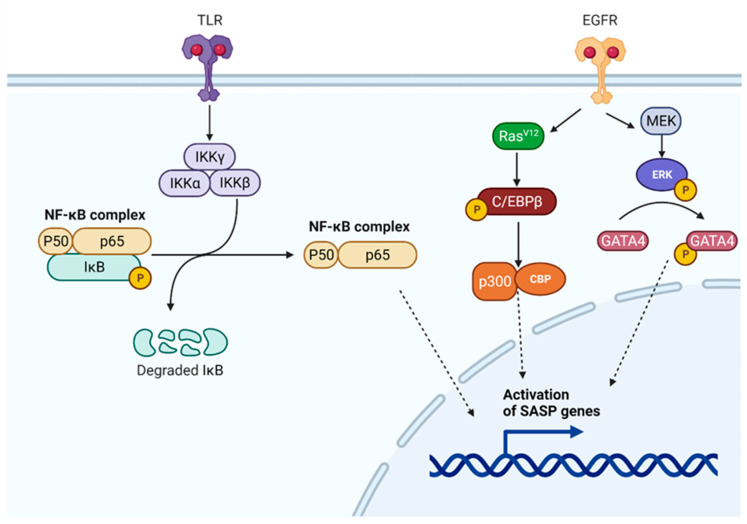
Main SASP regulatory pathways. SASP genes activation through the NF-κB transcription factor as the main regulator, in addition to C/EBPβ, p53, and GATA4.

One crucial SASP regulator that is frequently listed as the key transcription factor that controls SASP expression is the NF-κB regulatory pathway [89,93]. Dimer formation develops between NF-κB and DNA, regulating transcription, cytokine synthesis, and cell survival. In the cytoplasm, the common p65/p50 dimer is blocked by an inhibitory nuclear factor kappa B (IkB) protein (Figure 3). IkB kinase (IKK), which consists of the heterodimers IKK-α and IKK-β plus the regulatory protein IKKγ, also known as the essential modulator of NF-kappa-B (NEMO), activates NF-κB. Active IKK phosphorylates the IkB protein, resulting in its degradation to allow NF-κB to migrate to the nucleus and engage in transcriptional activity [95]. Various stimuli, including oxidative stress, diseases, growth factors, and proinflammatory cytokines, activate IKK. Alternatively, it can be turned on by mTOR via an interaction with IKK [95] or in the DDR, as the ATM/NEMO complex is exposed to the cytoplasm, in which NEMO binds and enables the IKKα/β heterodimer. At the same time, the p38 mitogen-activated protein kinase (MAPK) pathway can also affect NF-κB by activating the mitogen- and stress-activated protein kinases-1 and -2 (MSK1/MSK-2) that phosphorylate the p65 subunit of NF-κB [93]. In addition, the stimulator of the interferon genes (STING), which activates IRF3 and NF-κB, can become activated by cGAS via cGMP, causing senescence and SASP gene transcription via NF-κB activation. Additionally, STING can be triggered upon DNA damage by p53 and TNF receptor associated factor 6 (TRAF6), and therefore preferably activates NF-κB [89].

#### 7.1.2. C/EBPβ Regulatory Pathway

Members of the same transcription factor family, such as the CCAAT/enhancer binding proteins (C/EBPs), C/EBPβ, and C/EBPγ, may also have opposing effects on SASP regulation. C/EBPβ is an inducible transcription factor, similar to NF-κB, which has an activated regulatory pathway during OIS and controls the release of cytokines, such as IL-1β, IL-8, IL-6, GROα/CXCL1, and NAP2/CXCL7 [89]. C/EBP becomes phosphorylated within the presence of RasV12, which promotes the creation of homodimers, proceeding to further alterations and interaction with p300/CBP (Figure 3). The latter complex causes senescence and transcription of the SASP genes. C/EBPγ, on the other hand, decreases SASP genes expression in primary mouse fibroblasts through dimers formation with C/EBPβ. Elevated C/EBPγ has been linked to many human malignancies [94], whereas C/EBPβ is a requirement for senescence and can initiate it when upregulated [89].

#### 7.1.3. p53 and GATA4 Regulatory Pathways

A single pathway can potentially have distinct effects on different SASP factors. In particular, p53 stimulates the expression of some SASP factors while inhibiting the expression of others. In addition, it has the ability to inhibit p38MAPK, one of the key regulatory mechanisms involved in SASP formation [89]. Remarkably, the inhibitors of MDM2, which stop MDM2 from degrading p53 and thus stimulating p53 function, also decrease the expression of IL-6 and IL-1α [96]. This suggests that MDM2 inhibitors might be used as “senotherapy”, in addition to cancer treatment [97].

An additional key regulator of SASP is GATA4 (Figure 3), in which activation of ATM and ATR regulates GATA4, and this then stimulates IL-1α production and activates NF-κB [89,98]. Together, these two processes, IL-1α expression and NF-κB activation, have a significant influence on SASP expression via the positive feedback loop [99] and as key SASP-directing transcription factors [89,93].

### 7.2. Other SASP Regulatory Pathways

It is also known that epigenetic modifications, in the form of chromatin alterations, can also control SASP with the loss of repressive H3K27me3, known as a modification of the DNA packaging protein Histone H3, which has been associated with the downexpression of lamin B1 [100]. Other epigenetic alterations in senescent cells that enhance SASP include the histone chaperone HIRA, which leads to histone H3.3 variation deposit, as well as H4K16ac retention [89,101]. Acetylation of histones H3-K9 and H4-K16 has also been linked to reduced activity of SIRT1, which can control gene expression via deacetylation of various transcription factors or through silencing of chromatin scaffolds by histones deacetylation. SIRT1 inhibits IL-8 and IL-6 expression by targeting their promoter zones and deacetylating histones. SIRT1 translocates to sites of DNA damage as a response to DDR signaling in senescent cells, enabling depletion of previously silenced IL-6 and IL-8 genes, major SASP factors capable of promoting senescence in surrounding cells [102]. Chronic ATM activation leads to destruction of histone methyltransferases G9a and GLP and deletion of macroH2A.1, which is a tumor suppressor histone variation, from SASP chromatin, resulting in SASP gene derepression and induction [98].

By modifying chromatin architecture, high mobility group B2 (HMGB2) protein can also affect gene transcription due to its selective binding to SASP loci and upregulation in senescent cells, whereas its removal suppresses SASP gene expression [98]. A second alarmins member is the HMGB1, which rises in the serum and falls in the nucleus throughout aging and could also be a SASP regulator. Because it is produced extracellularly, it interacts with toll-like receptor (TLR) and activates the TLR/NF-κB pathway, whereas loss of nuclear HMG1B results in genetic instability and telomere impairment [89].

Alternatively, SASP can be induced through direct binding of bromodomain-containing protein 4 (BRD4) located closely to the critical SASP genes [98]. In senescent H-Ras V12-induced fibroblasts, MLL1 knockdown reduces the expression of the IL-1β gene, IL-1α, The knockdown of MLL1 decreases the production of certain factors, including IL-1α, IL-1β, IL-6, IL-8, MMP1, and MMP3 in H-Ras V12-induced senescent fibroblasts by inhibiting the ATM-NF-κB pathway [103].

The nuclear long non-coding RNA *MIR31HG* and the long noncoding RNA *SNHG29* are additional SASP regulators that independently control the production and secretion of a subset of SASP elements during BRAF- and H_2_O_2_-induced senescence, respectively. The intricacy of senescence and SASP regulation is illustrated by the lncRNA *MIR31HG*, which, depending on its cellular localization, can both induce and delay senescence [104]. When MIR31HG interacts with polycomb repressor complexes, which are overexpressed in different cancer types, it enhances the repression of p16/CDKN2A expression and promotes its cytoplasmic translocation. Similarly, upregulation of lncRNA *SNHG29* leads to a premature birth condition (PTB) via activation of mTOR/p53/p21 signaling. Senescence is promoted by SNHG29 overexpression, which also increases the production of SASPs, such as IL-8 and TNF-α, which have been shown to play a crucial role in PTB [105].

Retrotransposable long-interspersed element-1 (L1; LINE-1) activity has lately been revealed to be substantially active during replicative, oncogenic, and stress-induced premature senescence [106]. SIRT6 knockdown and aged animals both had enhanced L1 activity [107]. Transcription of L1 elements appears to occur in the presence of three regulators, namely, retinoblastoma protein 1 (RB1), forkhead box A1 (FOXA1), and three prime repair exonuclease 1 (TREX1), all of which show altered expression during senescence [106]. In contrast, the L1 promoter has been shown to be silenced by SIRT6. The cytosolic DNA sensor cGAS is triggered by cytoplasmic accumulation of L1/cDNA, which is thought to promote the innate immune response through type I interferon (IFN-I) responses [107]. While L1 activation along with the IFN-I response begins at a delayed stage after the initiation of senescence, it contributes significantly to the pro-inflammatory process and the generation of fully developed SASP [106], together with other age-related diseases [107].

As previously stated, several SASP components, particularly those produced early in SASP formation, such as IL-1α and IL-1β, promote and encourage continued SASP growth and release. The first one, IL-1β is expressed as a precursor in its mature stage [93], which induces senescence through phosphorylation of p38 [81]. As for the second, IL-α, it is activated as a mature precursor and obtained by cleavage of caspase 5 and caspase 11 [108]. It regulates the autocrine SASP signalling, promoting IL-6 and IL-8 production as part of a positive feedback loop involving interleukin-1 receptor (IL-1R), IL-1R-associated kinase (IRAK), NF-κB and C/EBPβ. IL-1α on the plasma membrane activates IL-1R [99] and triggers a signaling cascade that degrades IRAK1 and IkBα to allow nuclear transition of NF-κB [93,109]. IL-1α supports its own development through enhancing the level of intracellular Ca2+, thereby increasing calpain activity and IL-1α cleavage into a more mature form. Redox reaction can boost IL-1α expression even higher [93].

Apart from the positive feedback loop, senescent cells expressing high levels of SASP also have a negative feedback loop. When IL-1α levels are significantly elevated, the IL-1R pathway is activated, resulting in overexpression of miRNA-146a/b. The miRNA-146a/b induces a decrease in IRAK1 protein levels, which leads to a decrease in NF-κB activation and an underexpression of IL-6 and IL-8 genes, thus inhibiting excessive SASP function [110].

## 8. Prosurvival Pathways—Resistance to Apoptosis

In order to prevent senescent cells from undergoing apoptosis, they implement prevention mechanisms through activation of Bcl-2 family members (Bcl-2, Bcl-w, Bcl-xl, Bfl-1, and Mcl-1) or the p53-p21-serpin and phosphoinositide 3-kinase (PI3K)/AKT pathways. In addition, another way to resist apoptosis is through the ephrin-dependent receptor ligands ephrin-B1 and ephrin-B3 and plasminogen activator inhibitor-1 (PAI-1) [62], along with the hypoxia-inducible factor 1α (HIF-1α) and heat shock protein (HSP-90) pathways [111]. Yet, it is the cell type that determines which pro- apoptotic or anti-apoptotic protein is expressed [62].

The cell type and the level of DNA damage determine which cell goes into senescence and which goes into apoptosis, whereby p53 acts as a critical controller of this cellular response. Human diploid fibroblasts, for instance, exhibit partial senescence, as well as apoptosis, when exposed to H_2_O_2_. In apoptosis, they produce two times more p53 than in senescence, in which p21 inhibits p53-mediated apoptosis. Doxorubicin treatment produced two outcomes in SK-N-SH neuroblastoma and colorectal cancer cells: decreased p21 expression and thus apoptosis due to high doses, versus increased p21 expression, leading to senescence with low doses [57].

Other senescent cells with higher levels of Bax and Bcl-2, such as senescent epithelial cells (HUVEC), show greater potential for apoptosis [112]. Proteins of the Bcl-2 family have both pro-survival and pro-apoptotic actions. For example, the BH3 region of Bcl-2 family proteins is linked to apoptosis, and their single proteins can bind and block pro-survival agents (Bcl-2, Bcl-w, Bcl-xL, Bfl-1, Mcl-1) or trigger additional death agonists (Bax, Bak) [113].

A second theory proposes that BH3-only proteins block pro-survival compounds only by hindering them from binding to and blocking Bax or Bak. At the same time, p53 also promotes transcription of Bcl-2 genes or targets mitochondria on its own by interacting at the protein level with Bax and Bak. There is a role for the Bcl-2 family in the process of intrinsic apoptosis, mostly by modulating the permeability of the mitochondrial membrane, enabling the release of apoptogenic substances into the intermembrane region of the mitochondria. This activates Caspase-9, which then activates Caspases-3, -6 and -7, which break down the protein targets and produce morphological changes that lead to cellular death. Other proteins implicated in apoptosis are p73 and p63 [113].

Furthermore, the SERPIN family, SERPINB1, B4, B7, B9, and B13, was discovered to be upregulated in regular senescent human bronchial epithelial cells. IMMP2L is inhibited by SERPINB4, thus blocking the IMMP2L-AIF (apoptosis-inducing factor) pathway and conferring resistance to ROS-related apoptosis [76].

## 9. Other Features and Biomarkers of Senescent Cells

With respect to the morphology of senescent cells, they are flat, larger, and frequently multinucleated [60], having organelles that change in shape, weight, and function. The most widespread differences are an increased subcellular mass accompanied by functional deficiency and impaired signaling produced by the secretion of their metabolites. A possible reason for the elevated cell organelle count in senescent cells is a disruption of proteostasis and homeostasis. Increased cellular granularity is observed in many senescent cells, including lipofuscins in lysosomes, protein aggregations, secretory vesicles, and glycogen accumulations, among their constituents. The increase in the number of membrane organelles also increases lipogenesis. Sterol regulatory element-binding protein 1 (SREBP1), which is abundant in senescent cells and can induce senescence when overexpressed, is an excellent biomarker of the lipogenic state [77].

Accumulation of undegradable macromolecules and intracellular organelles in autophagic vacuoles increases cellular lysosomal content in senescent cells. As a consequence of the overloaded lysosomal content, subsequent digestion of macromolecules, proteins, organelles, and lipids is impaired, resulting in the formation of additional primary lysosomes. These lysosomes are likely to fuse with older lysosomes, resulting in an increase in the amount of larger lysosomes in senescent cells [60,84]. In addition, lysosomes in aging animals have a higher pH, despite the fact that digestion occurs in an acidic condition [74].

Senescence is a high-energy process requiring a constant supply of ATP. Due to the increased mitochondrial content per cell, senescent cells have an altered mitochondrial shape of a more complicated network, higher mitochondrial capacity, and increased respiration [114]. In senescent cells, dysfunctional mitochondria are more abundant; they do not synthesize ATP and instead generate higher levels of ROS. Their removal by autophagy is therefore necessary, but it is compromised by dysfunctional lysosomes, leading to their accumulation and high levels of ROS [74].

DNA, proteins, lipids, and enzymes, as well as other important molecular components, are also damaged by oxidative stress in senescent cells, together with other chemical alterations, such as glycation, oxidation, and cross-linking [60].

Senescent cells exhibit alterations in overall nuclear conformation, including the nucleolus, nuclear lamina, nuclear matrix, and nuclear bodies, as well as general hypomethylation [60].

The senescence process may involve spatial redistribution of heterochromatin into senescence-associated heterochromatin foci (SAHFs), the formation of which is promoted by the nuclear lamin B1 (LMNB1) knockdown-associated redistribution of perinuclear H3K9me3-positive heterochromatin, which can be prevented by efficient ectopic expression of LMNB1. In senescent cells, LMNB1 is largely reduced, while higher levels of binding were reported in some gene-rich regions, where H3K27me3 is elevated, but gene expression is suppressed [100]. Thus, alterations in the nuclear lamina may impact gene expression along with nuclear morphology. Loss of the B1 lamina is independent of the p38 MAPK/NF-κB, DDR, or ROS signaling pathways and can occur through activation of the p53 or p16^INK4^ tumor suppression pathways. Additionally, it is thought to be the result of higher mRNA instability induced by an unknown process [115]. The development of SAHFs is not limited solely to the reduction of LMNB1. However, it is also promoted by the pRB pathway, chromatin-associated non-histone proteins, and the histone chaperone HIRA and Asf1a and DNMT1 [116]. In addition, the connection of nucleoporin TPR (translocated promoter region, nuclear basket protein) with the nuclear pore complex in Ras-induced senescence of IMR90 cells has been shown to be responsible for SAHF formation and SAHF-associated SASP activation [117]. Specific antibodies can identify SAHF constituents (heterochromatin redistributed into senescence-associated heterochromatin foci), such as H3K9Me2, H3K9Me3, macroH2A, HP-1α, HP-1β, or HP-1γ, but SAHF can also be detected with DAPI staining. Importantly, mouse cells do not generate strong SAHF.

Nuclear foci called DNA segments with chromatin alterations reinforcing senescence (DNA-SCARS) form in senescent cells in response to DNA damage. Many proteins are recruited to remodeled chromatin, including the modified histone γH2AX that is increased in DNA-SCARS. DNA-SCARS are related to a variety of factors: promyelocytic leukemia nuclear bodies (PML), absence of DNA repair proteins, lack of replication protein A (RPA) and RAD51, absence of single-stranded DNA and DNA synthesis, and accumulation of activated versions of the DDR mediators, Chk2 and p53. These phenomena occur independently of p53, pRB, and other checkpoint and repair proteins, and they can be sustained for hours in living cells and for days or weeks in fixed cells. Although H2AX is required for DNA-SCARS stability, its absence impairs DNA-SCARS and decreases p53-dependent senescent growth arrest and IL-6 release. DNA SCARS can be found in senescent cells, such as replicative, H_2_O_2_-induced, or oncogenic RAS-senescent cells, but they are not restricted to senescent cells. Additionally, they can be found in proliferating p53-deficient cells that mimic premalignant lesions, as well as in tumor cells [118].

For the identification of DNA-SCARS, two assay methods can be used. In the first method, isolated cells are fixed and marked for DNA damage foci and PML bodies, following by an assessment of the amount and size of foci and co-localization of PML bodies. For the second technique, the abundance and persistence of DNA damage foci is monitored by observing the development and repair of DNA lesions in viable cells. This technique involves the development and production of a fluorescent fusion protein [60].

Other possible biomarkers for aging include lamin B1 loss [115], telomere stability, alterations in nuclear envelope, oxidative stress and BRAF, sirtuin, p66^SHC^ signaling, and lipofuscin [60]. Lipofuscin is an oxidized protein, and it is related to lipid and metal aggregation accumulating in the lysosomes of terminally-differentiated cells. Its presence has been detected in senescent cells, where it is colocalized with SA-βGal and can be stained with Sudan Black B. Importantly, since lipofuscin is not an enzyme, there is no need for a fresh sample; formalin-fixed tissue embedded in paraffin is suitable for use. This protein emits autofluorescence that can be revealed by fluorescence microscopy, or it can be stained with Nile Blue, Berlin Blue, Periodic Acid Schiff, and Ziehl-Neelsen [119].

α-Fucosidase, a lysosomal glycosidase that degrades glycoproteins, glycolipids, and oligosaccharides, can be revealed by X-Fuc and is an additional marker present in senescent cells independently of the type of stimuli, whether replicative, oncogenic, or TIS. It is suggested that the expression of α-fucosidase and senescence are closely related [120].

Finally, senescent cells can present certain proteins on their cell surface, among them the oxidative MDA modified vimentin, which was discovered to be present on the cell membrane of senescent primary human fibroblasts. Moreover, its increased secretion was observed in the plasma of senescence-accelerated mouse prone 8 (SAMP8) mice, suggesting that MDA-modified vimentin could potentially be used as a noninvasive senescence biomarker [121]. Another senescence biomarker is the dipeptidyl peptidase 4 (DPP4), a membrane-bound protein, which increases due to higher DPP4 gene transcription [122]. DPP4 is a protease that inactivates the gastrointestinal hormones incretins, particularly glucose-dependent insulinotropic peptide (GIP) and glucagon-like peptide-1 (GLP1). This impairs glucose homeostasis, which becomes weaker with age. In addition, it plays a role in SASP formation by activating the NF-κB signaling pathway in aged lymphocytes [122].

## 10. Interventions to Control Senescence

Any safe strategies able to delay or bypass senescence can have interesting outcomes in chronic disease prevention and may extend the healthy lifespan.

Metabolism is altered when cell age and the availability of nicotinamide adenine dinucleotide (NAD+) declines in the elderly [123]. NAD+ precursor rejuvenates muscle, neural, and melanocyte stem cells, preventing senescence in old mice through improvement of mitochondrial function [124]. Twelve-month old C57BL/6N wild-type mice treated orally with nicotinamide mononucleotide (NMN), a major NAD+ precursor, showed attenuation of age-related diseases with minimal side effects. Strikingly, NMN improved insulin sensitivity, blood lipid levels, ocular performance, lacrimation, and bone mineral density, and it reduced age-related weight gain [123]. NMN therapy has also been associated with an improved blood flow and endurance in older animals through promotion of sirtuin deacetylase SIRT1-mediated induction of capillary density, a result that is enhanced by exercise [125]. The potential of NMN for anti-ageing therapeutic strategy to delay chronic diseases by delaying senescence needs to be further explored in humans, although a single oral supplementation of NMN was shown to be safe, as it did not reveal significant clinical symptoms or alterations in health in men [126]. Interestingly, a mitochondria-targeted transmitter hydrogen-sulfide (H2S) was reported to delay endothelium senescence [127] and might have a general function of anti-vascular ageing playing a role via the regulation of endothelial NAD+ levels [125]

The effect of ketogenic diet [128,129], intermittent fasting [130], or exercise [131] on healthspan extension through metabolic regulation has been well documented, and ketogenesis-generated body beta-hydroxybutyrate has been recently revealed as a key player. This metabolite is described to be involved in the inhibition of vascular cell senescence [132], anti-inflammation [133], and immune activation by the formation and maintenance of CD8+ memory T cells [134]. More interestingly, beta-hydroxybutyrate prevents vascular senescence through hnrnpa1-mediated upregulation of Oct4 [132], the central player in reprogramming process towards pluripotency.

Additionally, fundamental studies showed that in vitro reprogramming was able to dedifferentiate adult cells with a cocktail of four reprogramming factors, Oct4, Sox2, c-Myc, and Klf4 (OSKM), except on senescent cells and with very low efficiency on cells from aged or progeric donors. We showed that an optimized reprogramming combination of six factors was able to convert, efficiently, many types of senescent cells and aged cells from extremely old people (centenarians) into pluripotent stem cells erasing the hallmarks of senescence and cellular aging, leading to a rejuvenated physiology and a reset proliferative capacity due to telomere lengthening through telomerase reactivation [135]. Interestingly, telomerase activation experiments were developed in a mouse model to bypass or escape RS patterns. Several separate investigations have shown that telomerase expression via genetic modification, virus administration, or chemical stimulation can greatly reduce age-related pathologies. As telomerase-based anti-aging methods, a telomerase activator and a telomerase gene therapy to avoid and decrease cellular senescence have been created. They have resulted in improved tissue regeneration and increased longevity without increasing cancer [136,137]. In recent years, the use of a high-capacity cytomegalovirus (CMV) vector as an intranasal and injectable gene therapy system to extend life has been shown to be an efficient and safe means of gene transfer for telomerase reverse transcriptase (TERT) protective factor. This therapy, in particular, increased glucose metabolism and muscular effectiveness while avoiding body mass reduction and baldness [138].

Similarly, transient reprogramming experiments in vitro and in vivo allowed us to improve cell physiology without dedifferentiation process and to increase longevity in mice by preventing senescence progression [139,140], even after a single transient reprogramming and applied early in life [141,142]. Cell reprogramming is consequently an additional suitable strategy to delay senescence.

## 11. Manipulating Secretion of Senescent Cell by Senomorphic Strategies

Extensive irreparable cell damage triggers a cell cycle arrest called cellular senescence. One of the most notable features of senescence is that senescent cells activate complex paracrine reactions defined as the SASP, in which they secrete many cytokines, chemokines, proteases, growth factors, and extracellular matrix remodeling factors. Factors secreted through senescent cells can play different roles upon the physiological setting.

### 11.1. Preventing the Expression and Secretion of the SASP

Reducing the harmful impacts of secretion from senescent cells without compromising their cycle arrest, as these processes are known to be independent of each other [143], revealed also interesting outcomes when eliminating senescent cells do not appear to be an effective technique.

Senescent cells display a strong proinflammatory secretion profile, which is widely consistent across various stages of senescence and biological sources [31,144,145,146]. SASP supports non-cell autonomous tumor suppression through cell cycle arrest of lesioned and malignant cells and by attracting immune cells to their destruction.

The specific secretome of senescent cells includes growth factors, pro-inflammatory cytokines, chemokines, and matrix remodeling metalloproteases, with a broad array of autocrine and paracrine impacts. Some of these factors are essential for resolving tissue damage, especially by immune activation and the support of growth arrest and differentiation [147,148], while others, notably the ones released for cellular growth, migration, and invasion, can be detrimental in disruption of tissue homeostasis and age-associated diseases formation [88] and cancer [149,150,151].

This complex secretome is directed by the activation of multiple signaling pathways, such as mitogen-activated protein kinase (MAPK) signaling, mammalian target of rapamycin (mTOR), GATA4/p62 intermediated autophagy, and the phosphoinositide 3 kinase (PI3K) pathways [152]. All of these cascades converge on the stimulation of NF-κB and C/EBPβ pathways, orchestrating the dynamics of the complex SASP secretome [144,146,153,154,155]. This huge variety of possible targets responsible for regulating the cascades, leading to the senescence associated secretory phenotype, incited the creation of different molecules and antibodies to interact either with NF-κB and C/EBPβ transcriptional activities at different levels [144,146,153,154,155], or with specific components of SASP to mitigate the deleterious effects of SASP. There is a compendium of available inhibitors with the ability to attenuate SASP effects in vivo and perhaps minimize the negative consequences of senescent cells.

Metformin, a type 2 diabetes treatment, inhibits NF-κB migration to the nucleus, limiting its expression levels [156]. Metformin reduces inflammatory reactions in mouse models, ameliorates several age-associated diseases, and increases longevity [157]. In addition to the consequences of metformin on diabetic patients, investigations for the impact of metformin on aging among humans are ongoing [158,159]. The TAME trial involves 3000 patients with at least one age-related disease, being aged 65–79 years, and will quantify a composite outcome that comprises cardiovascular events, cancer incidence, dementia, functional geriatric endpoints, and, finally, mortality. This trial represents the first case of intent to treat aging as a disease. It might pave the way for a revolution in the management of elderly subjects, with preventive medicine able to postpone age related pathologies development instead of curative medicine treating each disorder as an individual entity.

Additionally, the serine/threonine kinase mTOR is also upstream regulator of NF-κB, making specific inhibitors, such as rapamycin and it analogs, interesting targets to dampen senescence secretome indirectly by reducing NF-κB-transcriptional activity. Indeed, mTORs control the expression of the membrane-bound IL-1α. In human fibroblasts and breast cells, there are neutralizing antibodies against either IL-1α or its receptor, which are sufficient to reduce NF-κB activity [99]. Inhibition of mTOR by rapamycin and rapalogs can prevent SASP by lowering the expression of membrane bound IL-1α [109], thus lowering the transcriptional level of NF-κB [109]. Indeed, IL-1α/IL-1 receptor signal transduction is upstream of NF-κB, and utilising neutralizing antibodies against either IL-1 or its receptor is enough to suppress NF-κB enzymatic activity [99].

Recently discovered mTOR inhibitors containing everolimus, ridaforolimus, and temsirolimus are under investigations to evaluate their pharmacological characteristics that might target harmful secreted products from senescent cells [160,161]. mTOR is also involved in various cellular activities interacting with the MAPK pathway that promotes MAPKAPK2 translation [162], eventually leading to the activation of NF-κB and its translocation to the nucleus. Additionally, rapamycin prohibits senescent mouse fibroblasts [163] and suppresses MAPKAPK2 translation, causing degradation of secreted factors, including IL-1α and IL-8 [162]. Rapamycin also decreased the transcription level of IL-6, IL-1β, and VCAM-1 and inhibited the STAT3 pathway in lung WI-38 fibroblasts [164]. In addition, inhibitors acting on the MAPK pathway members were also identified as SASP modulators. In human senescent cells, the levels of mRNA and SASP factors released were reduced by the p38MAPK inhibitor SB203580, which decreased the transcriptional activity of NF-κB and the paracrine effect of factors secreted from these cells [165]. Likewise, IL-6 expression in human senescent fibroblasts is inhibited by next-generation p38MAPK inhibitors UR-13756 and BIRB 796. Additionally, senescent cells treated with the MAPKAPK2 inhibitors PF-3644022 and MK2.III have a reduced secretion phenotype [166].

Inhibition of JAK/STAT signaling by ruxolitinib is able to repress C/EBPβ transcriptional activity, reducing systemic inflammation and ameliorating fitness in old mice [92,167].

Other than the above-mentioned techniques, additional cellular senescence features can be used to create new therapeutics, markers, or diagnostic strategies. Senescent cells and associated secreted factors induce the release of important extracellular vesicles and exosomes, which contains different lipids, proteins, and microRNAs, thus reacting with the surrounding tissue and exerting significant effects on the immune regulation [168,169,170]. The exosomes released by the senescent secretome induce pro-tumorigenic roles mainly by stimulating extracellular vesicle-associated EphA2 binding to Ephrin-A1, thus promoting cancer cell proliferation [171]. Immune checkpoint ligands, such as B7-H3 protein, were found to be present in exosomes discharged by senescent cancer cells. It is, therefore, hypothesized that the detection and manipulation of extracellular vesicles secreted by aged cells can be applied in immunological therapeutic methodologies.

An additional strategy to control the secretion from senescent cells and the resulting immunologic response can be achieved by the regulation of cGAS-cGAMP-STING signaling pathway, a component of the innate immune system. Additionally, this pathway detects cytosolic chromatin fragments following DNA damage in senescent cells, stimulates type I interferons and further cytokines, and interferes with the auto-inflammatory diseases [172]. Deficiency of cGAS and STING in mice is correlated with a reduction in tissue inflammation following ionizing irradiation and an impaired immuno-surveillance of RAS oncogenes. Moreover, this pathway is triggered in human malignant cells and induces pro-inflammatory gene expression [172].

### 11.2. Targeting SASP Factors

While limiting inflammation is a potentially effective strategy to decrease senescent cells, non-cell autonomous effects, as well as therapy with anti-inflammatory medications, have the potential to create a wide range of negative effects [173]. These situations may preclude a protracted therapy regimen, which is required to maintain their favorable effects. The way to address this issue may be to tackle certain SASP elements, which may also give a better strategy to attenuate SASP’s negative impacts. Cytokines, which are well known constituents of SASP and include IL-6, IL-8, and matrix-remodeling proteases, might be potential targets. The use of neutralizing antibodies, which may be created using a variety of commonly produced monoclonal antibodies, is an appealing technique for blocking these compounds. Targeting IL-6 or its receptor, for example, might be accomplished using medications such as siltuximab or tocilizumab, which inhibit the cytokine or the receptor, respectively [174,175,176]. However, these compounds were not designed to be utilized in the setting of cellular senescence, and their consequences on SASP and aging must be investigated more before they may be used therapeutically as senescence regulators. Plasminogen activator inhibitor-1 (PAI-1), also called SERPINE1, is a key component of the SASP and a direct mediator of cellular senescence. In murine models of accelerated aging and, recently, in humans from an Amish community, genetic deficiency has been identified that protects against aging-like pathology and extends life span [177,178]. Targeted inhibition of PAI-1 with TM5441 showed similar activity in mice models, and a clinical trial with this molecule is ongoing to evaluate its effect on aging diseases by Dr. Toshio Miyata at Tohoku University in Japan.

## 12. Converting Senescent Cells into Apoptotic Cell by “Senolytics”

Senescent cells are similar to a double edge sword, with beneficial and detrimental effects.

Indeed, senescent cell secretions can prevent pre-malignant cells spreading and thus hinder tumorigenesis [179]. In other contexts, cell senescence encourages tissue repair and renewal [25,46]. Senescent cells may also influence morphogenesis accurately during embryo developmental phases in a damage-independent way [42,43]. Furthermore, aged cells can evoke organ restoration through self-elimination and recruiting immune phagocytic cells and mobilizing nearby progenitor cells.

Conversely, there are considerable negative effects of senescence; senescence can cause continuous damage to tissues and lead to a persistent pro-inflammatory microenvironment, which consequently leads to a variety of pathological conditions. Indeed, cellular senescence promotes a huge variety of age-related pathologies, such as cancer, obesity, type 2 diabetes, cardiovascular diseases, fibrosis, osteoarthritis, osteoporosis, sarcopenia, and neurological problems [4,5].

Due to the lack of a common aging hallmarks, senescent cells can be detected by searching for a range of biomarkers, which includes the upregulation of different cycle inhibitors, the exclusion of proliferative markers, the creation of particular heterochromatin domains, such as senescence-associated heterochromatin foci, and even a persistent activation of the DNA damage response. Because no gold standard exists to detect senescent cells, increased lysosomal SA-βGal continues to be one of the most useful indicators for cellular senescence, despite its absence of causal role in senescence process [180]. Cell cycle inhibitor p16 is an important marker of senescent cells and genetic ablation of p16 from aged/senescent cells in old or progeroid mouse models can improve cataracts, sarcopenia, cardiomyocyte, cancer development, renal glomerulosclerosis, osteoarthritis, atherosclerosis, hypertrophy, tumorigenesis, adipose atrophy, and tau-based disorder [6,10,14,15,16,78], thus increasing animal lifespan [15,16].

Consequently, the major challenge to translating these recent finding into safe and efficient senotherapies lies in suppressing the negative aspects of cellular senescence while eventually enhancing the positive ones, taking into account the potentially negative repercussions of removing senescent cells.

### 12.1. Senescence-Associated β-Galactosidase Activity to Target Senescent Cells

Increased lysosomal SA-βGal continues to be one of the most useful markers of cellular aging [38], and its activity was envisaged to be used to kill senescent cells. A targeted delivery system employing mesoporous silica nanoparticles covered with galacto-oligosaccharides was created based on the ability of SA-βGal to enzymatically digest galactose preferentially in senescent cells, leading to the release of cytotoxic drugs content of the particles [181]. Nevertheless, it is recognized that some other cells within the human body, such as macrophages, exhibit high β-galactosidase activity under certain conditions [182], therefore elevated β-galactosidase activity is not absolutely specific of senescent cells. Strikingly, another senescence associated lysosomal alpha–Fucosidase (SA-alpha-Fuc), was not further generalized to detect senescent cells, whereas it has been described as a more robust senescence marker than SAβ-Gal. It might be used for nanoparticule engineering as an optimized option [183]. Another option to increase selectivity might be to tether the nanoparticles to antibodies against identified senescent surface markers [184]. Senolytic methods relying on SAβ-gal activity have not yet been evaluated in vivo, and additional research is needed to assess the efficacy of such therapies. Combining senotherapies, such as mTOR inhibitors with other senescence-modulating drugs, can allow reduction of drug dose and their off-target effects [185]. Additionally, the direct intake can precisely attack the senescent tissues or organs while reducing the exposure of unaffected ones.

Another method for pathway-based senolytic development was using high throughput approaches involving modulations of SA-βGal activity as a primary readout. This caused the discovery of HSP90 proteins, a family of ubiquitously expressed molecular chaperones, as a novel class of targets [186]. They can stimulate cellular survival through AKT or ERK maintenance [187,188], members of signaling pathways upregulated in ageing [189,190]. The anti-apoptotic PI3K/AKT pathway is downregulated by HSP90 inhibitors, reducing senescence indicators in different cell lines. A HSP90 inhibitor, 17-DMAG, has been clinically tested in a variety of solid tumors and lymphomas, and it decreased the expression level of p16. In vivo, administration of the inhibitor 17-DMAG to progeria mouse model reduced the senescence signature, postponed multiple age-associated clinical signs, and extended health span [186,191]. Similarly, the ATM pathway, a member of the DNA damage response signaling, activated in senescence also regulate SA-βGal activity senescence by controlling lysosomal acidification. KU-60019, an inhibitor of ATM, was discovered as a powerful anti-senescence factor [74]. ATM-treating senescent fibroblasts with KU-60019 reduced SA-βGal activity, cleared damaged mitochondria, and reprogrammed their metabolism. Moreover, KU-60019 improved epidermal wound healing, and the blockage of ATM function also reduced senescence; thus, KU-60019 is thought to be a viable target for treating age-associated pathologies [74].

Metabolic reprogramming is essential for senescent cells to adapt them to the elevated energy needs of the senescent cycle, involving proteotoxic stress due to massive secretion from senescent cells, increased oxidative stress leading to misfolded or harmful proteins, and expanded endoplasmic reticulum stress [192]. Consequently, metabolically targeted drugs might also be promising, but it remains to be seen whether these medications can attain adequate selectivity between senescent and non-senescent cells.

### 12.2. Inhibiting Pro-Survival Pathways with Senolytics

Due to the fact that senescence and apoptosis have been considered as alternate cellular fates in the presence of injury and stress, pro-apoptotic cellular alterations are frequently actively anti-senescent, whereas senescent cells are extremely resistive to apoptosis. Multiple senescent molecular pathways substantially contribute to improved survival [57,193].

The best known pro-survival pathways are BCL-2 proteins family, as well as the p53 and PI3K/AKT pathways. Targeting these pathways can be exploited to remove senescent cells from damaged or aged tissues [57,193].

p53, the most commonly mutated cancer tumor suppressor gene, is a central component of the senescence program [194]. p53 is stabilized and accumulates in response to DNA damage [22,195,196], and the p53/p21 prosurvival pathway is a decision point between transient cell cycle arrest, senescence, and apoptosis [194]. Consequently p53/p21 axis was investigated as a potential target for development of senolytics [197,198]. FOXO4 transcription factors that favor cell cycle arrest and senescence was described to interact with p53 preventing p53-induced apoptosis [199]. Interfering in p53’s direct interaction with the transcription factor FOXO4 leads to the release of p53 from the nucleus and induction of cell-intrinsic apoptosis. Thus, a D-retro-inverso peptomimetic of FOXO4 interfering with the interaction FOXO4/p53 was designed to restore in vitro p53-induced apoptosis in human senescent fibroblasts [198]. In aged mice, the FOXO peptomimetic neutralized doxorubicin-mediated senescence and chemotoxicity, limiting hepatotoxicity and restoring lost weight and renal capacity in progeroid and naturally aged mice [79,198]. The FOXO4 peptide’s senolytic action is promising, but also other peptides or peptide-mimetic compounds might form the foundation for future senolytic medication discovery.

The BCL-2 proteins are recognized for their regulatory effect in programmed cell death, and they are frequently dysregulated in cancer, as well as in diverse autoimmune and degenerative diseases [200]. A specific inhibitor of BCL-2, BCL-xL, and BCL-W is Navitoclax (previously known as ABT-263). Navitoclax selectively induces apoptosis in either replicative, oncogene-induced, or irradiated senescence triggered in various cell types (human umbilical vein epithelial, human lung fibroblasts and mouse embryonic fibroblasts), but surprisingly not in primary human pre-adipocytes [201]. A study in irradiated mice revealed that Navitoclax depletes senescent cells and rejuvenates bone marrow and muscle hematopoietic stem cells and thus reduces early aging of the hematopoietic system [13]. In addition, Navitoclax diminished the secretion of many factors from senescent murine lungs, such as p16^INK4A^ and TNFα, and it also lowered the risk of post-traumatic osteoarthritis by selectively eliminating senescent cells from the articular cartilage and synovial membrane [10]. There was also an amelioration of age-associated symptoms in a study focused on senescent osteoblast progenitors [202]. This drug also reduced secretion from senescent cells in aged mice, attenuating osteoclastogenesis in bone marrow stromal cell culture [202]. A structural relative of Navitoclax, ABT-737, was tested, in the presence of MEFs and IMR 90 human fetal lung fibroblasts characterized by the high expression of BCL-2, BCL-W, and BCL-xL anti-apoptotic proteins, following various senescence-promoting stimuli [12]. ABT-737 cleared senescent cells from the lungs and the epidermis of irradiated mice and improved proliferation of hair follicle stem cells. Despite their great outcome, Navitoclax and ABT-737 are considered dangerous due to their toxic effect on neutrophils and platelets, which could restrict their clinical advancement [203]. Nevertheless, second-generation inhibitors against BCL-xL, A1331852, and A1155463 promoted a targeted apoptosis of aged HUVEC, as well as IMR90 cells, while again sparing senescent human pre-adipocytes [204]. Since human pre-adipocytes appear impervious to BCL2 inhibitors, there is likely a heterogeneity in the intrinsic cellular senescence pathways [201]. Further, the third generation of senolytics targeting the BCL-2 family is focused on the BH4 domain, which is found on all the pro-survival family members (BCL-2, BCL-xL, BCL-W, MCL-1, and BFL-1) and which is essential for their anti-apoptotic action [205].

Another typical pro-survival factor that senescent cells depend on is the Myeloid Cell Leukemia 1 (Mcl-1). Because Mcl-1 is the most overexpressed anti-apoptotic gene in senescent cancer cells, including Bcl-2-negative senescent tumor cells, pharmacologically suppressing Mcl-1 can ultimately eradicate senescent prostate cancer cells, preventing the spread of the tumor and metastases. Mcl-1 inhibitors are considered an extremely potent class of senolytics. While Navitoclax minimizes the occurrence of metastasis in vivo, S63845, a Mcl-1 inhibitor, completely eradicates both senescent tumor cells and metastasis [206].

Recently, it was found that chimeric antigen receptor (CAR) T cells could have a potential senolytic effect by targeting senescent cells and killing them by induction of apoptosis via the extrinsic pathway. Induction of apoptosis has been shown to be induced after association of CAR T cells with Bcl-2 family inhibitors, such as ABT-737 [207]. Senescent cells strongly express the urokinase-type plasminogen activator receptor (uPAR) on their surface, which is subsequently detected by uPAR-specific CAR T cells, inducing senescent cell elimination in vitro and in vivo. Targeting uPAR by CAR T cells increased the longevity of mice with lung adenocarcinoma and improved liver fibrosis conditions in mice [208].

### 12.3. Combination of Senolytics with Chemotherapeutic Agents

Other molecules with potential senolytic activities were identified because they improve the outcome of chemotherapy (inducing senescence) in various cancer models. This is also the case of the BCL-xL specific inhibitors, A1331852 and A1155463, which improve the outcome of chemotherapy (inducing senescence) in mouse models of ovarian cancer, breast cancer, and non-small-cell lung cancer without risking the cytotoxic chemotherapy-induced neutropenia seen with Navitoclax [209]. Piperlongumine, a natural senolytic agent isolated from trees of the genus Piper, was initially found to inhibit tumour growth in a xenograft mouse model of tumor. Piperlongumine induces apoptosis in senescent cells triggered by oncogenes, radioactive ionization, or exhaustive replication [210]. Similarly, Panobinostat, a histone deacetylase (HDAC) inhibitor, has a senolytic effect in non-small cell lung cancer and in head and neck squamous cell carcinoma cell lines that had already been treated with Cisplatin or Taxol cytotoxins [211]. Panobinostat increases the activity of caspase-3 and -7 and reduces the expression of Bcl-xL in chemotherapy stimulated senescent cells. More recently, a group of senolytic compounds, which are cardiovascular glycosides, targeting cellular membrane Na+/K+-ATPase pumps, were described to render senescent cells more susceptible to apoptosis due to a resulting electrochemical gradient imbalance. These complexes were effective ex vivo in pre-neoplastic senescent cells and in vivo in cases of lung fibrosis, treatment-triggered senescence, and old wild type mice [212,213].

An effective combination for clearing out senescent cells both in vitro and in vivo is the coupling of Dasatinib and Quercetin. Dasatinib is a tyrosine kinase inhibitor blocking the SRC, c-KIT, ephrin receptors, and different kinases. Quercetin is a flavonoid that targets various kinases and receptors and blocks the PI3K-AKT pathway. This drug duo is effective on many different kinds of senescent cells [11,214]. Indeed, Dasatinib plus Quercetin (D+Q) ameliorated cardiovascular capacity and reduced p16 and senescence-associated b-galactosidase expression in limb-irradiated aged mice [214]. Frequent administration of D+Q in progeroid mice increased survival by slowing down age-associated symptoms and diseases. The drug duo also reduced physical disorders in naturally aged mice and in senescent preadipocyte-transplanted mice. D+Q reduced senescent cell counts and pro-inflammatory cytokine secretion in human fat tissue taken from obese people [215]. It is also believed that cellular senescence leads to idiopathic pulmonary fibrosis and D+Q reduced senescence and fibrosis in primary alveolar epithelial type II cells taken from fibrotic mouse lungs [216,217]. Furthermore, this drug mixture destroyed senescent cells and improved fibrotic lung disorder in a bleomycin-damage mouse model [7]. D+Q also cleared senescent cells from blood vessels, thus improving the vascular environment related to age-associated vascular diseases [11]. Aortic plaques were also reduced in a hyper-cholesterolaemic mouse model of atherosclerosis chronically treated with this combination. A study also revealed that D+Q not only decreases cellular senescence, but also necroptosis in Sod1 knockout mice by reducing the availability of senescent cells that secrete SASP factors, such as TNF-α, thereby lowering the level of inflammation and retarding the development of numerous age-related diseases and aging [218]. In conclusion, D+Q are highly compatible senolytic drugs, which have been widely verified in clinical trials of various age-related diseases.

Nevertheless, cellular senescence has been found to have a detrimental role during liver transplantation, with opposing DCR2 functions in cholangiocytes and hepatocytes. Intriguingly, Ferreira-Gonzalez et al. demonstrated in 2022 that constant infusion of a D+Q combination of senolytics attenuated cold-induced biliary injury, hindered liver degradation, and improved overall liver preservation, making senolytics an effective strategy for pre-liver transplantation [219].

Additionally, a range of flavonoid polyphenols other than quercetin have been investigated for their senolytic effects. Fisetin reduced senescence signatures in various tissues in both progeric and physiologically aged mice, and treatment of normally aged mice with fisetin recovered tissue homeostasis, lowered age-related impairment, and increased longevity [220]. Prospective therapeutic results are needed for a first proof-of-concept of combination drugs.

### 12.4. PCC1, a New Type of Phytochemical Senolytic

Many fruits and vegetables contain ubiquitous phenolic compounds, known for their antioxidant and anti-free radical activity and therefore used in recent years as anti-carcinogenic, anti-inflammatory, anti-viral, and anti-bacterial pharmaceutical drugs. These compounds are made up of flavan-3-ols chains, catechin and epicatechin, connected with each other via C4–C6 and C4–C8 inter-flavonoid bonds, with gallate esters [221]. Grape seed extracts (GSE) contains an important polyphenolic component, the flavonoid procyanidin (PCC1), which, due to its senotherapeutic activity, is able to eliminate senescent cells and ameliorate age related pathologies. At low concentrations, this B-type trimeric epicatechin showed an inhibition of SASP factors secretion, while it displayed a deterioration of senescent cells at higher doses by its apoptotic activity. Previous studies revealed that PCC1 can reduce oxidative damage, suppress inflammation, and induce apoptosis in tumor cells. It acts by downregulating senescent-2 while upregulating BAX and activating caspases 3 and 9 and thus releases cytochrome c and disrupts the mitochondrial membrane, which protects from cancer by initiating apoptosis. Senotherapeutic activity of PCC1 has been proven on different cells undergoing senescence through either replicative senescence, oncogene induced senescence (HRAS^G12V^), or stress-induced senescence following a predetermined starting dose of 50 µM, which selectively kills senescent cells without affecting non-senescent cells [222].

PCC1 was also found to eliminate p16-positive senescent cells as effectively as ABT-263, in addition to its ability to increase tumor suppression, inhibit resistance to chemotherapy, and ameliorate physical dysfunction. Both D+Q combination and PCC1 can eliminate all three types of senescent cells, but only PCC1 has no toxic effect on proliferating cells. This is in addition to a high specificity and efficiency against senescent cells compared to ABT-263, dasatinib, quercetin, and fisetin [222].

### 12.5. Senolysis Effect by Targeting CRYAB with 25-Hydroxycholesterol

Because senolytics are known to eliminate senescent cells in vivo by targeting genes responsible for senescence survival, many pharmaceutical studies have focused on finding these genes and have discovered CRYAB, an important senescence-related gene and a potential senolysis target, through single-cell RNA sequencing. 25-Hydroxycholesterol (25-HC), an endogenous metabolite of cholesterol, has been classified as a senolytic due to its ability to disrupt CRYAB and thus fight age-related pathologies induced by cellular senescence through targeting and eliminating senescent cells in both human and mice tissues [223]. 25-HC is an oxysterol acting in cholesterol homeostasis, increasing inflammatory reaction and having anti-viral activity, but it also plays a role in the pathogenesis of neurodegenerative diseases and atherosclerosis [224,225]. 25-HC was also demonstrated to suppress autophagy dysfunction through the mTOR/STING pathway in PC12 cells [224] and to prevent SASRS-COV-2 from replicating through membrane cholesterol depletion. It also blocks the secretion of SASP factors and, in particular, IL-6 in skeletal muscles [223]. Other oxysterols, such as 24-HC and 27-HC, should be examined for their possible senolysis activity.

However, as the action of each senolytic can be restricted when senescence is triggered in various ways, senolytic drugs are unique to a given therapy. The senolytic effects of certain drugs, such as GT, ABT263, and the Akt inhibitor MK2206, upon the induction of cellular senescence in androgen-sensitive prostate cancer (PCa) LNCaP cells, were demonstrated by Pungsrinont et al. in 2020 to be different [226]. In androgen receptor (AR) agonist-induced cellular senescent cells, the pro-survival signaling is more active than in an AR antagonist. Additionally, depending on the type of AR ligand, treatment with GT or MK2206 displayed varied senolytic actions, but ABT263 lacked senolytic potential.

## 13. Immune System Mediated Interventions to Clear Senescent Cell

### 13.1. Immunosurveillance of Senescent Cells

**Figure 4:**
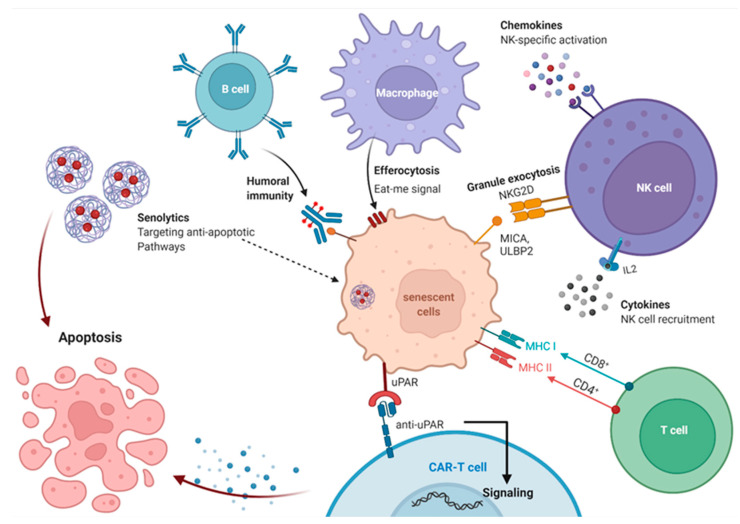
Clearance of senescent cells through immune system activation or apoptosis induction via senolytics. Distinctive ligands generated by senescent cells, including MICA, ULBP2, MHC I, and MHC II recognized by immune cells, such as NK cells, T cells, macrophages, and CAR-T cells. B cells eliminate senescent cells through a humoral antibody immunity.

Senescent cells are frequently removed by the immune system as part of a complicated mechanism that involves both the innate and adaptive immune systems (Figure 4). As a result, numerous immune cell types, including macrophages, neutrophils, natural killer cells (NK), and CD4+ T cells, are involved in the evacuation of senescent cells. However, the precise processes permitting is still elusive. SASP chemokines and cytokines are thought to function as chemo-attractants for NK and several immune cells [227,228]. Furthermore, senescent cells highly express adhesion molecules, including intracellular adhesion molecule-1 (ICAM-1), vascular cell adhesion molecule-1 (VCAM-1), and selectins, which may have a role in senescent cell removal [228,229].

Ligands of the activating NK cell receptors, group 2 (NKG2D), one of which is MHC class I polypeptide-related sequence A (MICA) and the other second being UL16 binding protein 2 (ULBP2), are increased and displayed on the cellular membrane in conditions of DNA damage, as well as oncogene-induced or replicative senescence. This enables NK cells to recognize and eliminate specific targets. DDR promotes the production of ULBP2, whereas ERK may influence MICA and ULBP2 levels via promoting mRNA stability by decreasing the expression miRNA activity. Because NKG2D receptors are found on a fraction of T-cells, immunosurveillance may be a mutual role of NK and T-cells. The abundance of NKG2D receptors on NK cells does not vary with age, although NK activity is known to decline [228]. Furthermore, the membrane-bound malondialdehyde (MDA)-vimentin might be an additional age-related cell specific element identified by innate immunity in a senescent cell removal process that progressively weakened with age [121].

It is not yet known why senescent cells build up within life, but the immune system plays a major function in the elimination of aged cells, and the increase in senescent cells in tissues is a consequence of the age-dependent decline of the immune system. It was discovered that senescent dermal fibroblasts contain the non-classical MHC class 1b molecule (HLA-E), which interferes with the inhibitory receptor NKG2A expressing NK and CD8+ cells, which in turn decreases the clearance of senescent cells. The expression of HLA-E is increased in senescent cells coming from elderly skin. The expression of SASP-derived proinflammatory cytokines, especially IL-6, is controlled by p38 MAP kinase signaling. Similarly, NKG2A activation of CD8+ T cells rises during aging. Alternatively, the number of NKG2A+ NK may decline as people age, yet their expression is generally high, explaining why NKG2A blockage has a more significant impact on NK than on CD8+ cells. If both NKG2D and NKG2A are expressed, the documented NKG2D-dependent elimination of senescent cells may be compromised. In vitro inhibition of the NKG2A/HLA-E connection improves the immunological reaction to senescent cells. As a result, it is hypothesized that this pathway contributes to the buildup of senescent cells throughout aging [227].

While many senescent cells evade apoptosis, the immune system, which includes both adaptive and innate immune cells, has a significant impact on eliminating senescent cells [230]. The elimination of senescent cells is induced via CD4+ T cells, as well as monocytes and macrophages, through a mechanism called senescence surveillance [231]. However, with ageing, human immune cells are more prone to become senescent. It affects several immune system activities, which gradually decrease with age, a mechanism called « immunosenescence » [232]. Accordingly, an improved immunosurveillance ability was revealed in heathy supercentenarians people due to a higher presence of CD4+ cytotoxic T lymphocytes, and of the inhibitory receptor natural killer group 2A (NKG2A)-positive CD8+ T cells. This might explain their increased longevity and their ability to regularly eliminate senescent cells [233].

If senescent cell accumulation in aged tissues is due in large part to declining immune function with age, boosting the immune system may lead to successful removal of cells from aged tissues, as demonstrated by the use of an immunostimulator to mediate NK cell-mediated removal of senescent cells in livers fibrosis [26]. Consequently, senescence immunotherapy strategy recently emerged as a senolytic substitute in the protection and treatment of senescence and chronic conditions [231].

### 13.2. Immune Boosting Strategies to Improve Elimination of Senescent Cells

As the immune system becomes less efficient with age, it is likely that a therapeutic boosting of immune responses can be useful for improving senescent cell elimination.

Senescent cells are naturally immunogenic and are subject to context-dependent immune surveillance mechanisms. Senescence induction by p53 remodeling stimuli an innate immune reaction typically consists of invading leukocytes, neutrophils, macrophages, and NK cells in a model of hepatocellular carcinoma [33] and age-associated hepatic stellate cells enhance their own NK cell-mediated eradication in cases of liver fibrosis [26]. Senescence induction by NRASG12V expression in hepatocytes leads to a dual release of both innate and adoptive immunological response, as CD4+ T cells collaborate with monocytes/macrophages to remove senescent hepatocytes [35]. This procedure is mediated by stimulating natural killer cell receptor NKG2D, which detects ligands on the external membrane of stressed, infected, or injured cells. Senescent cells increase the expression of the immunological recognition protein NKG2D, which is normally absent from the membrane of healthy cells [228]. Additionally, NKG2D was needed for natural killer cell-induced senescent cell elimination to protect from fibrotic liver formation [228]. Therefore, the expression of NKG2D may be useful in immunotherapy strategies.

Boosting the immune clearance of senescent cells can also be achieved by adapting cancer vaccine strategies. Even though no universal biomarker of cellular senescence is known, the contact between immune and senescent cells might induce an immunological response to undetected antigens.

Senescence vaccines would consist of isolating senescence-specific antigens and exposing them to antigen presenting cells and dendritic cells. Therefore, the dendritic cells would express these antigens on their cellular surface, which would flag them to T cells. The interaction response between T cells and these antigens provokes the activation and differentiation of T cells and eventually kills targetted senescent cells [231].

A useful strategy is the production of cells able to detect particular components of the senescent secretome. This was proved in creating cells harboring a chimeric IL-6 receptor (IL6Rchi), which produces a Ca^2+^ signal when in contact with IL-6, an important component of the senescent secretome [234].

### 13.3. Antibody-Dependent Cell-Mediated Cytotoxicity Eliminates Senescent Cells

Adding to the immune enhancing methods, adapting immunotherapy strategies normally dedicated to cancer treatments might reveal important outcomes for ageing disease interventions. Blocking the immune checkpoint Programmed Death 1 (PD-1) is an example of a potent anticancer therapy. which can help alleviate clinical symptoms in an Alzheimer’s disease mouse model [235].

A great example of an anti-cancer therapy is illustrated by using engineered immune cells, such as chimeric antigen receptor T cells targeting individual molecules on tumor cells. Similarly, senescent cells can be targeted via engineered T cells expressing NKG2D chimeric antigen receptor, which detects NKG2D ligands on target cell surfaces [236,237]. The lack of selective senescence markers is a hurdle in immune-targeting techniques.

Senescent cells are mainly secured from NK cell-mediated cytotoxicity due to increased expression of the decoy receptor 2, DcR2 [238]. DcR2 prevents TNF-related apoptosis-inducing ligand (TRAIL) from activating death receptors 4 and 5 (DR4/DR5), limiting the killing process to perforin- and granzyme-mediated pathways. Targeting strategies to accelerate this process, such as by inhibiting DcR2, may render senescent cells more susceptible to immune defense and immunotherapy-based targeting.

As mentioned earlier, natural killer cells detect and destroy senescent cells through NKG2D ligand membrane expression [228]. These NKG2D ligands are also expressed by cancer cells, which make them an important target for cancer immunotherapeutic strategies [239] and consequently for senescent cell clearance.

DPP4 was discovered to be more abundant on the surface of fibroblasts that had undergone RS than on normal cells [122]. Treating senescent cells with an anti-DPP4 antibody induces an NK cell favorable killing in an antibody-dependent cell-mediated cytotoxicity assay [122,240].

Besides, recent studies are working on identifying particular epitopes and membrane receptors related to senescent cells in order to develop antibody-based therapies or screening factors that are more selective and have fewer negative effects. Both DEP1 and B2MG [241], in addition to DCR2 [242], are examples of overly expressed identified markers in senescent cells, but these markers also appeared among other cells and damaged tissues.

Recently, the use of CD9 receptors associated with increased SA-βGal activity is being tested in an in vitro study as a dual nanoparticle targeting better drug delivery to senescent cells [184]. Similarly, a senescent lung fibroblasts marker was discovered, a membrane-bound vimentin oxidized type [121]. In senescent fibroblasts, humoral innate immunity may recognize and attack the oxidized precursor of vimentin. Altogether, these findings therefore suggested a possible immunotherapeutic approach targeting cellular senescence. However, further in vivo studies are required to prove the clinical applicability of these experimental approaches.

One can believe that this approach might ultimately be used in healthy people to postpone aging. We should re-evaluate the best approaches to translate beneficial pre-clinical findings into well developed and effective therapeutic processes. Several approaches targeting senescent cells currently face to drug development challenges, including cost and time, to bring a potent and widely used anti-aging treatment to the pharmaceutical market, while being effective, safe, and tolerable, which is taken into consideration in the initial phases of development.

The major unanswered question in this discipline is the efficacy of various senotherapies in diverse human tissues. The majority of current data on such therapies come from in vitro cell culture research, which does not represent the setting of a pathology and may slightly replicate the condition in vivo. Pharmacological in vivo efficacy was frequently investigated in mice models, many of which are partially related to aging and age-associated diseases. As a result, prior to serotherapies’ implementation in clinical trials, their significance to particular human diseases must be thoroughly explored. Furthermore, existing techniques target a broad population of senescent cells, despite the fact that senescent cells are very diverse. The varying sensitivity of cell subpopulations to a particular therapy may result from differences in the cells’ origin, the stimulus of senescence, or the disease environment. Therefore, it is reasonable to assume that certain therapies will be more appropriate than others for a specific tissue or age-associated condition. Thus, the idea of considering senotherapies as a pan-antisenescence therapy is rather confusing and should be reconsidered. This will require evaluation of the impact of each therapy on various cell populations while employing the most appropriate model for every disease condition to which the drug can be used.

Because of the physiological importance of senescent cells and the adverse effects of current senolytics, safety concerns may arise when adopting senescence-targeted therapies. Upon systemic administration, many senolytics, particularly BCL-2 family inhibitors, are toxic to non-senescent cells [203]. In addition, many senescence epitopes may be displayed to some extent in non-senescent cells, such as cellular stress or secreting cells, in the case of immunotherapy. There are several alternative solutions to this issue. Drugs, for example, could be delivered directly to the target zone. More importantly, synergistic techniques based on the delivery of senescent cell-specific senolytic agents could have a synchronous effect, allowing the delivery of smaller, less aggressive doses with increased efficacy and safety. Another avenue for improving specific targeting is to modify the drug so that it becomes active only after exposure to an enzyme produced by senescent cells. In addition to the negative impact of the targeted component, decreased safety may result from a likely interaction with the positive activities of senescence. Although the senescence process is implicated in embryogenesis, tissue repair, rejuvenation, and tumor suppression, the impact of senescence targeting by senolytic therapy on such processes is yet unknown [42,43,243,244,245,246]. Current senolytics induce apoptosis of senescent cells during limited drug exposures, which can be applied for a short period of time. New therapies must take this into account and be administered in a spatially and temporally regulated manner, in addition to well designed studies to avoid failure. First-wave senotherapies must be carefully selected for indications that may include an unusual age-related condition for which standard treatment is only symptomatic and not therapeutic. The function of cellular senescence in the pathophysiology of each condition or disorder must be established, and the underlying mechanisms must be well understood. The existence of appropriate preclinical models will enable the assessment of the risks and benefits of each therapy. Specific biomarkers of senescence or disease indicators to be used for prognosis and prediction are also critical to the success of clinical investigations. The lack of good senescence markers continues to limit fundamental research, where quantitative measurements are restricted to ex vivo cellular examination. Novel senescence biomarkers are needed to study senescence levels non-invasively in tissues so that participants can be selected based on their senescence status. Many factors can contribute to the success of experimental trials, including scientific evidence, experimental parameters, and individualized patient conditions. Altogether, cellular senescence is a complex process involving both advantageous and disadvantageous outcomes. Senotherapies have the potential to inhibit adverse outcomes and cure a wide range of age-related diseases, but proper administration is necessary to ensure both efficiency and safety. The importance of understanding the processes of potential adverse reactions when determining the method of administration and concentration will accelerate the clinical development of senolytic drugs and provide new opportunities for improving human lifespan and health.

## 14. Clinical Targeting of Senescence in Age-Associated Disorders

Age-related diseases and their corresponding social burden are increasing. Age-associated immune senescent modifications might lead to a decrease in the immune system, chronic inflammation, in addition to weakness, chronic disease, and loss of function among old people [247]. Translating approaches that target senescent cells into clinical practice could have a major impact on curing a range of diseases that occur with age and perhaps change our perspective of ageing.

Preclinical studies showed that the clearance of aged cells can improve and reverse multiple age-associated diseases [41,248,249], giving hope and promises to develop new therapeutics for these critical disorders. For this reason, clinical trials targeting senescent cells are under development.

Due to impressive preliminary results, many trials are focusing on D+Q as a potential bi-therapy to some age-associated diseases and mainly chronic kidney disease (NCT02848131), idiopathic pulmonary fibrosis (NCT02874989), and in cases of hematopoietic stem cell transplant, presenting a high risk of premature ageing (NCT02652052) (Table 1).

Persons diagnosed with activated PI3K Delta syndrome have a dominantly mutated PI3K catalytic subunit p110δ, causing senescence of T cells and immunodeficiency [250]. Leniolisib, a new effective and selective oral PI3Kδ (CDZ173) inhibitor, was able to reduce senescent T cells and to lower the inflammatory markers in clinical trials formed of six patients (NCT02435173); also, an extension study is progressing (NCT02859727). Further procedures are being developed, which include more trials to detect the effect of plasma transfer from healthy young male donors to patients over 40, assessing the ability to reverse epigenetic and other senescent markers (NCT03353597). Many factors can be evaluated from blood and skin biopsies, such as the DNA methylation level and other epigenetic alterations, the length of telomeres, as well as renal, pulmonary, cognitive, and muscle strength variations, in addition to IGF-1 and p16 expression.

The early findings of a phase two experiment examining twelve weeks of senolytical therapy of knee osteoarthritis showed failure to meet the expected outcome, with the complete data still to be reported (UNITY, 2021). The phase 2 clinical study, UBX0101, in patients with painful osteoarthritis of the knee, was conducted by Unity Biotechnology. Many explanations could be given as to why the experiment failed: the strategy is not practical for this condition, maybe the drug dosage was wrong, or the inclusion criteria should have been narrower or broader. All of this aside, clinical trials of senescence-reducing therapies are in their earliest stages and need to be further explored. However, recently, a red-hot anti-aging approach almost passed its first test after 14 volunteers took drugs meant to kill off old, toxic cells present in their bodies. They suffered from a fatal, hard-to-treat lung condition called idiopathic pulmonary fibrosis. Idiopathic pulmonary fibrosis is a lethal condition characterized by age-related markers, such as DNA damage, inflammation, telomere attrition, oxidative stress, and, most significantly, cellular senescence [251]. Studies in mice with idiopathic pulmonary fibrosis show that the senolytic agents D+Q have a beneficial impact on pulmonary functioning [7]. At the same time, a study among patients with idiopathic pulmonary fibrosis, which primarily investigated the overall safety of this treatment, indicates that D+Q also has beneficial implications for physical function in humans [252]. For three weeks, fourteen patients suffering from idiopathic pulmonary fibrosis received D+Q treatment, and the results showed not only significant, but also clinically meaningful gains, in physical function. This analysis showed no effect on lung capacity, clinical chemistry, or frailty. As for the effects on SASP and proinflammatory markers, it was not entirely conclusive, but it showed some correlated changes [252].

Diabetes and renal disease are associated with an increased senescent cell load, particularly in adipose tissue. A clinical experiment examined the effects of D+Q on senescent human adipocytes [253], in which 11 patients with diabetes and kidney disease received D+Q for three days. Results showed a substantial decrease in markers of senescent cells, as well as significantly lower numbers of senescent cells in adipose tissue. Ultimately, the study confirmed the postulated hypothesis by showing that D+Q significantly decreased circulating SASP components in the patients.

Moreover, numerous studies have been conducted on MitoTam, a mitochondria-targeted tamoxifen that has been shown to be a novel senolytic that selectively activates senescent cell death by decreasing mitochondrial membrane potential and preventing mitochondrial oxidative phosphorylation (OXPHOS), thereby improving mitochondrial stability [254]. Further than this, MitoTam has been shown to effectively destroy a variety of renal cancer cells and inhibit renal carcinomas in a rodent model via involvement of mitochondria, resulting in reduced tumorigenesis (EudraCT 2017-004441-25) and providing hope for future phase 2 trials [255]. Finally, MitoTam also appears to have the potential to be an anti-diabetic agent, helping to tackle one of the most prevalent pandemics, obesity and type 2 diabetes mellitus [256].

At present, 20 clinical trials on senolytics are ongoing. Two of the studies targeting frailty and aging (AFFIRM studies), comparing fisetin to placebo as a control group, are almost halfway complete. One of these studies is in elderly women with severe frailty who have a walking speed of less than 0.6 m per second, which is a significant indicator of a 2-year survival rate of about 50%. A pilot study in Alzheimer’s disease (ALSENLITE) has been initiated, along with a multicenter, double-blinded, placebo-controlled study in Alzheimer’s patients (SToMP-AD) and a study in renal disease, along with a nearly completed study in bone marrow transplant patients (HTSS). Patients requiring bone marrow transplantation receive high doses of chemotherapy and radiation to knock out their immune system prior to transplantation, which unfortunately can cause aging. A number of bone marrow transplant recipients have been found to develop accelerating signs of aging after three to five years. Therefore, this trial will test if elimination of senescent cells could mitigate this accelerated aging condition in which individuals develop many pathologies including diabetes, cognitive decline, secondary non-related cancers, and atherosclerosis with myocardial infarctions and strokes.

All trials are in preparation for a bigger double-blinded, placebo-controlled study. The NIH-funded study of age-related osteoporosis is nearly completed, with over half the participants recruited. Another trial by the Office of Naval Research supported a clinical trial on osteoarthritis, a condition associated with the accumulation of senescent cells in the joints of the knees. Additionally, a number of studies on coronaviruses and their consequences are still currently active or have been initiated (COVID-FIS, COVID-FISETIN, COVFIS-HOME).

## 15. Future Perspectives

The abnormal and permanent accumulation of various senescent cells causes aging and the emergence of chronic diseases. Cellular senescence regulation is crucial for healthy aging, as it also has short-term benefits. There are multiple clinical molecules and senolytics to treat aging and chronic diseases related to cellular senescence.

Even though immunological elimination of specific senescent cells is a novel and challenging technique for anti-aging and treatment of several chronic conditions, it is not costless and has negative effects. For clinical translation of this new technology, certain inquiries need to be conducted, including: (1) both preclinical and clinical research, identification of appropriate biomarkers or molecular mechanisms for personalized aging; (2) discovery of distinct senescent cell antigens or ligands for immune monitoring; and (3) incorporation of both sexes into the study due to the gender variations in aging and chronic disorders. Senotherapy can lead to adverse reactions and harm tissue homeostasis and function. This may be avoided by applying senotherapy in a temporary and discontinuous manner if it preserves the therapeutic effects of delaying aging.

In this review, we present certain important approaches to control cellular senescence, some of which were investigated and confirmed on animal models with aging-related diseases. A subset is now in clinical trials. Our main focus here is on senolytic drugs and manipulating the immune surveillance of senescent cells. Importantly, several clinical approaches targeting senescent cells might have a significant effect on treating some age-related pathologies and on increasing lifespan through an aging delay in the damaged tissues and organs. However, some critical risks should be taken into consideration for a cautious clinical translation of senescence-targeted therapeutic methodologies.

Much of the data on senotherapies were from human cell cultures. The new approaches promote senescence through various stressors and controlling mechanisms and in various cell types. The induction of senescence in specific cell types from specific tissues through either replicative stress, oncogene activation, irradiation, carcinogens, damaging molecules, and many others helps determine the key elements and signaling pathways involved [257]. There are many different types of senescent cells, and consequently, the subtypes can have multiple vulnerabilities to senotherapies and senoprobes.

Thus, it is very crucial to clinically solve the correct questions as to how drug 1 affects drug 2 and in which conditions, by assessing different models accurately representing the human condition. Senescent cells are known to autonomously inhibit the spread of damaged and pre-malignant cells, and thus, they form an essential tumor-resistance barrier [179]. It was observed that the accumulation of mutations in the p53–p21 and the p16-Rb pathways are correlated with the majority of human cancer cells and among different senescence-related genes [258]. Additionally, escape from or reverting senescence contribute to carcinogenesis [259]; correspondingly, it was discovered that a family of therapy-induced senescent cancer cells can develop functional and phenotypic stemness characteristics, causing cell cycle entrance, self-renewal ability, and a higher tumor phenotypic aggressiveness [260]. Despite their many advantages, senescence-focussed approaches should be translated from relevant preclinical models with extreme caution due to the beneficial and harmful roles that senescence exerts, depending on the pathological situation [4].

Moreover, the secretome of senescent cells is importantly involved in recruiting T cells and macrophages, which facilitates immuno-surveillance in the case of liver precancerous lesions [231]. However, the senescent secretome might also intrinsically induce chronic inflammation and tumor progression [150]. In fact, secretion from senescent cells helps in repairing damaged and injured tissues. An example of this comes from senescent-activated stellate cells, which limit liver fibrosis by increasing immuno-surveillance and reducing secretion of extracellular matrix components, apart from extracellular matrix degrading enzymes [26].

The same kind of situation occurs when senescent fibroblasts assemble In healing cutaneous wounds of granulated tissues and activate anti-fibrotic genes [45]. Mouse studies revealed that senescence contributes to fibrotic pulmonary disease models, as fibrosis was reduced and lung function improved when these mice were treated with senolytics, anti-inflammatory molecules, and therapeutic nanoparticles [7,217,261].

The contradictory roles of senescent cells, as inhibitor and enhancer of fibrosis, in mouse models of human fibrotic diseases highlights the need for more comprehensive knowledge on the intrinsic mechanisms and pathways causing cell cycle arrest and the specific elements released by senescent cells prior to initiating clinical trials in humans.

## 16. Conclusions

As life expectancy has increased, so have the chances of contracting age-related diseases, such as cancer, so understanding the aging process is key to improving life. During aging in mice, as well as in humans, senescent cells accumulate, leading to tissue dysfunction and to different age-related pathologies. Preclinical studies in mice proved that eliminating senescent cells can improve and even reverse the pathological appearance of several disorders; however, many obstacles must be overcome to guarantee a perfectly strategic translation to the clinic.

When cells are damaged and stressed, senescence or apoptosis cell fates are safeguard mechanisms with bright and dark side. In some circumstances, it might be more interesting for tissue fitness to maintain senescence and mitigate the deleterious effect of the SASP with senomorphic strategies and, in other situations, elimination of senescent cells using senolytic therapy could be more advantageous clinically. As there are various molecular mechanisms behind the resistance of senescent cells to apoptosis, for each age-related disease it will be important to identify their signaling pathways, specific secretomes, and senescent arrest contributors. Additionally, a more stringent selection of better optimized senotherapies is required to improve efficacy and reduce toxicity. Bearing in mind inter-patient individuality, and that the aging process with its related disorders, is multifactorial and complex, it is most likely that the next generation of anti-aging health interventions will be specialized to depend on specific biomarkers that inform a clear risk–benefit ratio.

Many senotherapeutic and immunological studies of age-associated pathologies are currently in clinical trial. It is likely that, ultimately in the long term, immune cell-mediated elimination of senescent cells will be key to reduce senescent cell burden. Expeditious clinical advancement will thus depend on a better understanding of senescent cells and their interaction with the immune system.

This critical era that we are going through will allow us to develop anti-senescent therapies, which might have a major effect on personalized ‘precision’ medicine. These advances in tissue repair mechanisms and regeneration will likely further increase human healthspan and longevity.

## Figures and Tables

**Table 1:** Ongoing and planned clinical trials using senolytic drug interventions.

Intervention/Treatment	Disease/Condition	Outcome Measures	Eligible Age	Phase	Study ID (https://clinicaltrials.gov/, accessed on 24 June 2022)
**Dasatinib + Quercetin**	Idiopathic pulmonary fibrosis patients	Improvements in physical function	≥50	Phase I	NCT02874989
	Diabetes and kidney dysfunction	Decrease in senescence markers	50–80	Phase I	NCT02848131
	Alzheimer	Change in cellular senescence blood marker	≥65	Phase II	NCT04063124
	Skeletal disease	Skeletal health improvement	≥70	Phase II	NCT04313634
	Chronic kidney disease	Senescent cells proportional change	40–80	Phase II	NCT02848131
	Stem cell transplant	Frailty level	≥18	Recruiting	NCT02652052
**Fisetin**	Frailty, inflammation	Decrease in blood inflammation markers	≥70	Phase II	NCT03430037
	Osteoarthritis, knee	Change in levels of proinflammatory markers associated with senescence	40–80	Phase I	NCT04210986
	COVID-19	Change in COVID-19 severity	≥65	Phase II	NCT04771611
**Dasatinib + Quercetin; Fisetin**	Childhood cancer and Frailty	Senescence reduction and frailty improvement	≥18	Phase II	NCT04733534
**UBX0101**	Osteoarthritis, knee	Safety and efficacy level	40–85	Phase II	NCT04129944
**UBX1325**	Diabetic Macular Edema Neovascular age-related macular degeneration	Gain in visual acuity	≥50	Phase I	NCT04537884
**UBX1967** **UBX 2050**	Macular degeneration	-	-	-	Planned
**CDZ173**	APDS/PASLI	Senescent T cells reduction	12–75	Phase II and III	NCT02435173 (Extension study NCT02859727)
**Tamoxifen (MitoTam)**	Solid metastatic tumours	Maximum tolerated dose identification Safety level High efficacy against renal cell carcinoma	18–75	Phase I/Ib	EudraCT 2017-004441-25

## Data Availability

Not applicable.
